# Changes in LDL-cholesterol levels following aromatase inhibitor treatment in early postmenopausal breast cancer

**DOI:** 10.1007/s10654-025-01228-7

**Published:** 2025-05-21

**Authors:** Marie Lund, Giulia Corn, Maj-Britt Jensen, Tonny Petersen, Kim Dalhoff, Bent Ejlertsen, Lars Køber, Jan Wohlfahrt, Mads Melbye

**Affiliations:** 1 Department of Epidemiology Research, Statens Serum Institut, Artillerivej 5, 2300 Copenhagen S, Denmark; 2 Department of Clinical Pharmacology, Bispebjerg and Frederiksberg Hospital and University of Copenhagen, Copenhagen, Denmark; 3 Department of Clinical Medicine, Faculty of Health and Medical Sciences, University of Copenhagen, Copenhagen, Denmark; 4 Danish Cancer Institute, Statistics and Data Analysis, Copenhagen, Denmark; 5 Danish Breast Cancer Cooperative Group, Copenhagen University Hospital - Rigshospitalet, Copenhagen, Denmark; 6 Department of Clinical Oncology, Rigshospitalet and University of Copenhagen, Copenhagen, Denmark; 7 Danish Breast Cancer Cooperative Group, Rigshospitalet and University of Copenhagen, Copenhagen, Denmark; 8 Department of Cardiology, Rigshospitalet and University of Copenhagen, Copenhagen, Denmark; 9 Danish Cancer Institute, Cancer Epidemiology and Surveillance, Copenhagen, Denmark; 10 Department of Clinical Medicine, Aalborg University, Aalborg, Denmark; 11 K.G. Jebsen Center for Genetic Epidemiology, Norwegian University of Science and Technology, Trondheim, Norway; 12 Department of Pediatrics, Stanford University School of Medicine, Stanford, CA USA; 13 Danish Cancer Institute, Copenhagen, Denmark

**Keywords:** Early breast cancer, Endocrine treatment, Aromatase inhibitors, Lipids, LDL-cholesterol

## Abstract

There is an unsettled concern that treatment with aromatase inhibitors (AIT) may adversely affect lipid-levels. In light of the improved survival of women with breast cancer and increased risk of atherosclerotic cardiovascular disease in older people, unfavorable effects on lipid-levels may represent a significant health concern for this group of patients. We used linked data from nationwide registries, including a clinical breast cancer database with information about allocated and dispensed AIT. Based on these, we investigated changes in plasma lipid-levels (primary outcome: low-density lipoprotein (LDL)-cholesterol, secondary outcomes: high-density lipoprotein (HDL)-cholesterol, total cholesterol, and triglycerides) following AIT in a nationwide cohort of postmenopausal women with early breast cancer, Denmark, 2009–2020. Included women had at least one LDL-cholesterol measurement before and after breast cancer diagnosis. Exposure was allocated and dispensed AIT as compared with not allocated to and no dispensed AIT. Outcome was the adjusted difference in lipid-level-change (from before to after breast cancer diagnosis) according to AIT. Among 10,461 women, there were 22,693 pre-breast cancer LDL-cholesterol measurements and 42,750 post-breast cancer LDL-cholesterol measurements. Overall, 7919 of the women were exposed to AIT and 2542 women were unexposed. For AIT exposed, the LDL-cholesterol-change was − 0.16 mmol/L (mM), and for unexposed, − 0.15 mM, respectively. The corresponding adjusted difference in LDL-cholesterol change for AIT exposed versus unexposed was − 0.03 mM (95% CI − 0.07 to 0.003). We found similar results in analysis of secondary outcomes. This study does not support the concern that AIT adversely affects lipid-levels.

**Supplementary Information:**

The online version contains supplementary material available at 10.1007/s10654-025-01228-7.

## Introduction

For estrogen receptor positive breast cancer, aromatase inhibitor treatment (AIT) is used as a single agent in postmenopausal women and in premenopausal women in combination with ovarian function suppression or ablation [1, 2]. AIT may be associated with an increased risk of adverse plasma lipid levels; however, results do not show any definitive patterns of plasma lipoprotein variations [3]. It is important to clarify the association since low-density lipoproteins are causal in the development of atherosclerotic cardiovascular disease (ASCVD) [4]. Adverse effects on lipid levels could therefore represent a compelling general health concern given the improved breast cancer survival [5] and higher risk of ASCVD in older people [6].

Estrogens affect vasodilation and vascular remodeling thereby impairing atherosclerosis [7]. Estrogen levels are reduced at menopause [8]. The risk of cardiovascular disease (CVD) increases after menopause [9]; in part thought to be explained by estrogen deficiency and mediated by unfavorable changes in the lipid profile [8, 10]. Peripheral conversion of androgen into estrogen by the aromatase enzyme is central in the post-menopausal production of estrogen [11, 12], and by inhibiting this, AIT suppress estrogen levels [13]. It may therefore be speculated that an adverse effect of AIT on lipid-levels is mediated by estrogen depletion. The frequencies of hyperlipidemia are variably reported in the different AIT labels [14–16] which may in part be explained by different monitoring of lipid-levels across the different trials preceding market authorization [17–19]. In the BIG 1,98 trial (adjuvant letrozole), no increase was found in the median percent change in cholesterol from baseline [17]. The label for anastrozole reports no clinically significant changes in lipid-levels (low-density lipoprotein cholesterol [LDL-cholesterol], high-density lipoprotein cholesterol [HDL-cholesterol], triglycerides and total cholesterol) during treatment [14]. Generalizability to patients treated in everyday clinical practice, is nevertheless limited by trial ineligibility criteria including various definitions of comorbidities i.e. including cardiovascular comorbidity [18–20].

In terms of isolating an effect of AIT on lipid-levels, comparison with tamoxifen comes with the limitation of being a selective estrogen receptor modulator i.e. tamoxifen may in some tissues act like estrogen and in other tissues block estrogen actions [21]. Tamoxifen may therefore have estrogen agonist effects on plasma lipids and has been associated with beneficial effects on the lipid profile [3]. However, a systematic review [3] of the effects of AIT on plasma lipoproteins revealed only three relatively small studies (between 147 and 411 included individuals) with a placebo-based comparator group and all of the studies exclude women with (various definitions of) disease affecting the lipid levels [22–24]. Substudies of the MA.17-trial (5 years letrozole vs. placebo after ∼5 years tamoxifen) and ATENA-trial (5 years exemestane after 5–7 years tamoxifen) did not convincingly suggest an overall detrimental effect of AIT on plasma lipid-levels [22, 23]. Notably, in both studies [22, 23] the treatment with AIT followed a treatment course of tamoxifen which could imply a carry-over effect. Two small uncontrolled studies in women with metastatic breast cancer provide conflicting results, and a recent Dutch cross-sectional study finds no association between AIT and dyslipidemia, but none of the studies included a comparator group [25–27].

Thus, it remains unclear whether AIT per se is associated with an unfavorable effect on the lipid profile versus a non-tamoxifen-based comparator group. In this nationwide cohort study, we utilize information about a cohort of postmenopausal women with early breast cancer from the clinical database of the Danish Breast Cancer Group (DBCG) to investigate changes in plasma lipid levels following initiation of AIT. To mitigate the concern of using tamoxifen as a potentially cardioprotective comparator, we designed the study with no endocrine therapy as comparator.

## Methods

### Data sources

The main data source for this study was the clinical database of the Danish Breast Cancer Cooperative Group (DBCG-database) [28]. In brief, adjuvant breast cancer treatment in Denmark is determined by nationwide guidelines according to patient and tumor characteristics. Guidance is provided to the treating physicians through automated algorithms that are built into the database assigning specific treatment protocols to the patients. For reasons for no inclusion in a treatment protocol, see Table S1. Patients assigned to the low-risk group receive no systemic treatment; for criteria for being assigned to the low- and high-risk groups, respectively, since 2009, see Figure S1. For the high-risk group, the specific combinations of treatment modalities have varied over the years i.e. various combinations of chemotherapy, endocrine therapy, biological therapy, radiation therapy and treatment with bisphosphonates [28, 29]. Information about allocated and dispensed treatment is part of the DBCG-database. Except for patients belonging to the low risk group, adjuvant AIT (letrozole) has been recommended to postmenopausal, ER-positive women since 2009 (since April 2010 including also treatment recommendation if 1–9% ER positive except since 2019, if non-luminal subtype as determined by mRNA expression of 50 genes (PAM50)) [29, 30].

In addition to the DBCG-database, we based the study on individual patient information from various Danish nationwide health and administrative registers linked using unique personal identifiers (Table S2). For detailed definitions of inclusion and exclusion criteria, study population, exposure, outcome and covariates described below, see Table S3.

### Study population

All postmenopausal women diagnosed with early breast cancer, January 1, 2009 to December 31, 2020, according to the DBCG-database were eligible for inclusion. Date of breast cancer diagnosis (defined as surgery date or biopsy date if neoadjuvant treatment) constituted the index date. We excluded women who at the index date: had a diagnosis of other primary cancer excluding non-melanoma skin cancer, were not included in a treatment protocol according to the DBCG-database, had less than 2 years of residency in Denmark; and women who in the period between index date and start of the time window for included post-breast cancer lipid measurements (defined below) died, emigrated, had tamoxifen dispensed or who deviated from the allocated treatment protocol (i.e. no dispensed AIT if allocated to AIT, or dispensed AIT if not allocated to AIT). Furthermore, we required for cohort members to have at least one measured pre- and post-breast cancer LDL-cholesterol level. For a graphical illustration of the study design, see Figure S2.

### Exposure to aromatase inhibitor treatment

As main exposure, we studied women who were allocated to endocrine treatment (which for postmenopausal patients was AIT during the entire study period) and who had AIT dispensed (termed ‘Use of AIT’). AIT exposure was ascertained in the period from index date until 3 months after or end of chemotherapy, whichever came last. The comparison group included women not allocated to AIT and to whom AIT was not dispensed (termed ‘No use of AIT’). As secondary exposures, we updated the use of AIT during the time window for included post-breast cancer lipid measurements (defined below) comparing current and past with never use, stratified use of AIT by time since treatment cessation, and studied allocation to AIT vs. no allocation to AIT as exposure.

### Lipid levels

We included both pre- and post-breast cancer lipid levels. The time window for included pre-breast cancer lipid measurements included all measurements in the 2 years preceding the index date. The time window for included post-breast cancer lipid measurements started 3 months after surgery for breast cancer or end of chemotherapy whichever came last and ended on the date of emigration, death, December 31, 2021 (end of study period), or 5 years after the start date of the time window, whichever came first. All available measurements in the two time windows were included in the models. The primary and secondary lipid level outcomes were LDL-cholesterol, HDL-cholesterol, total cholesterol, and triglycerides, respectively.

### Statistical analysis

**Fig. 1:**
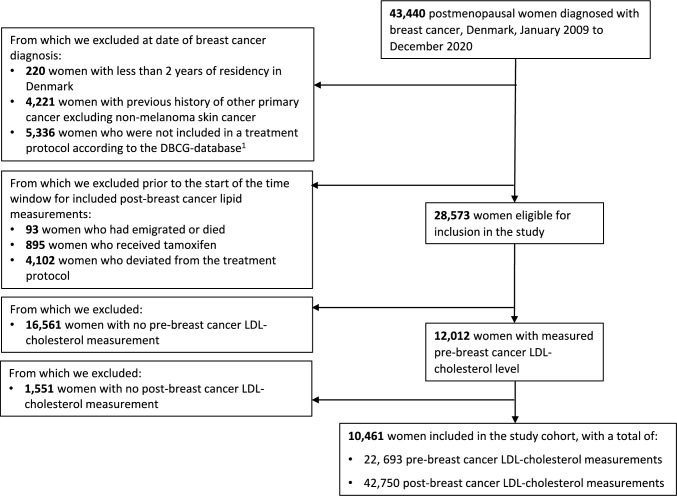
Cohort attrition. Abbreviations: DBCG-database the clinical database of the Danish Breast Cancer Group; LDL, low-density lipoprotein. ^1^For reasons for no inclusion in a breast cancer treatment protocol, see Table S1

To estimate the difference in change in lipid-levels before and after breast cancer diagnosis according to use of AIT, we used a linear mixed-effects model with the change between the (observed) post-breast cancer lipid level and the pre-breast cancer lipid level (predicted at index date) as outcome, overall and according to subgroups. The main model included, as fixed effects, use of AIT, demographic characteristics (age [2-year intervals], calendar period [1-year intervals], education, region of residence, disposable household income, and cohabitory status), tumor characteristics (laterality, size, histology and malignancy grade, lymph node involvement, human epidermal growth factor receptor 2-status), other anti-cancer treatments (type of surgery, chemotherapy, and radiotherapy), the interaction between laterality and radiotherapy, and the time of lipid measurement; and a random intercept, to account for the inter-individual variability. Each woman contributed with a number of observations equal to the number of available post-breast cancer lipid measurements and all observations from the same women were weighted for the inverse of the probability of having a post-breast cancer LDL-cholesterol measurement. The probability of having a post-breast cancer LDL-cholesterol measurement was calculated as the product between the probability of having a post-breast cancer LDL-cholesterol measurement given that a pre-breast cancer LDL-cholesterol measurement was registered and the probability of having a pre-breast cancer LDL-cholesterol measurement; both these probabilities were estimated using logistic regression. The pre-breast cancer lipid level at index date was predicted for each individual from a linear mixed-effect model with the pre-breast cancer lipid level as outcome including several fixed effects and one random intercept. The statistical models, the included covariates and number of women contributing to each model are described in further detail in Supplementary Methods, Table S4 and Fig. 1.

In the main analyses, missing values accounted for less than 3% for all variables and were imputed using mode imputation within protocol strata except for AIT for which women with unknown registration were classified as non-users. In sensitivity analyses of the primary outcome using the same statistical approach, we evaluated selection bias, alternative definitions of exclusion criteria and exposure, and performed analyses with further adjustments and additional criteria for end of the time window for included post-breast cancer LDL-cholesterol measurements.

We performed all analyses using SAS version 9.4 (SAS Institute, Inc., Cary, NC, USA), procedure MIXED.

## Results

After application of exclusion criteria, there were 10,461 women in the final study population, i.e. who had at least one LDL-cholesterol measurement before and after breast cancer diagnosis (Fig. 1). There were 7919 women with use of AIT and 2542 women with no use of AIT. Distribution of the main covariates are presented in Table 1 and additional covariates in Table S5. Age, socioeconomic variables and medical history were evenly distributed among the two groups, including similar proportions with use of lipid-lowering drugs (LLD, approx. 39%) and CVD (approx. 53%). Reflecting treatment protocols over time, there were more women with no use of AIT in the early study period (2009–2012), who had small tumors (< = 1 cm), who had no involvement of lymph nodes and who were treated with chemotherapy.

**Table 1:** Distribution of main covariates according to use of AIT

	Use of AIT		No use of AIT	
	*n*	%	*n*	%
Overall	7919	100.0%	2542	100.0%
**Demographic characteristics at index date**^a^				
Age (median, IQR)	68(62,75)		67(62,72)	
*Calendar period*				
2009–2012	1374	17.4%	629	24.7%
2013–2016	2748	34.7%	816	32.1%
2017–2020	3797	47.9%	1097	43.2%
*Disposable household income*				
1st quartile	2344	29.6%	725	28.5%
2nd quartile	2618	33.1%	855	33.6%
3rd quartile	1671	21.1%	563	22.1%
4th quartile	1286	16.2%	399	15.7%
*Education*^*b*^				
< 10 years	2664	33.6%	887	34.9%
10–12 years	3179	40.1%	1011	39.8%
13–15 years	1749	22.1%	559	22.0%
≥ 15 years	327	4.1%	85	3.3%
*Co-habitation*				
Yes	4808	60.7%	1556	61.2%
No	3111	39.3%	986	38.8%
**Tumor characteristics**				
*Laterality*^*b*^				
Left	4083	51.6%	1306	51.4%
Right	3836	48.4%	1236	48.6%
*Tumor size*^*b*^				
< = 1 cm	851	10.7%	1119	44.0%
> 1–2 cm	4337	54.8%	827	32.5%
> 2–5 cm	2500	31.6%	551	21.7%
> 5 cm	231	2.9%	45	1.8%
*Histology and grade*^*b*^				
Lobular—Grades I-III^c^	1179	14.9%	172	6.8%
Ductal—Grade I	1530	19.3%	894	35.2%
Ductal—Grade II	3663	46.3%	254	10.0%
Ductal—Grade III	1065	13.4%	800	31.5%
Other histology	482	6.1%	422	16.6%
*Lymph node involvement*^*b*^				
Negative	4907	62.0%	2117	83.3%
Positive^d^	3012	38.0%	425	16.7%
1–3 positive lymph nodes	2231	28.2%	239	9.4%
4–9 positive lymph nodes	444	5.6%	76	3.0%
≥ 10 positive lymph nodes	245	3.1%	43	1.7%
*HER2-status*^*b*^				
Positive	718	9.1%	353	13.9%
Negative	7201	90.9%	2189	86.1%
**Treatment characteristics**				
*Type of primary surgery*^*b*^				
Mastectomy	2179	27.5%	556	21.9%
Breast conserving surgery	5740	72.5%	1986	78.1%
*Radiotherapy (allocation)*				
Yes	6673	84.3%	2148	84.5%
No	1246	15.7%	394	15.5%
*Chemotherapy*^*b*^				
Yes	1650	20.8%	1218	47.9%
No	6269	79.2%	1324	52.1%
*Neoadjuvant treatment*				
Yes	227	2.9%	188	7.4%
No	7692	97.1%	2354	92.6%

AIT, aromatase inhibitor treatment; HER2, human epidermal growth factor receptor 2; IQR, interquartile range.

‘Use of AIT’ is defined as allocated and dispensed treatment with AIT, and ‘No use of AIT’ is defined as no allocated and no dispensed treatment with AIT; both as recorded in the clinical database of the Danish Breast Cancer Group (see Table S3 for further definitions).

^a^Defined as date of breast cancer diagnosis (defined as surgery date or biopsy date if neoadjuvant treatment) as recorded in the clinical database of the Danish Breast Cancer Group.

^b^Missing information was imputed as described in Table S3 (education = 142, laterality < 5, tumor size = 44, histology and grade = 83, lymph node involvement = 55, HER2-status = 121, type of primary surgery = 28, chemotherapy = 287).

^c^Lobular grade I, II and III have been grouped due to small numbers. When adjusting, lobular tumors are grouped according to grade I, II and III.

^d^Numbers of women within the categories of number of positive lymph nodes do not summarize to 3012 since for some of the women number of positive lymph nodes were unknown (primarily patients who had received neoadjuvant treatment).

The median length of the time window for included post-breast cancer lipid measurements was 4.1 years (interquartile range [IQR 2.8–4.5]) for women with use of AIT and 4.1 years (IQR 2.8–4.6) for women with no use of AIT. For women with use of AIT, we included 17,220 pre-breast cancer LDL-cholesterol measurements (median pr. women of 2) and 32,540 post-breast cancer LDL-cholesterol measurements (median pr. women of 3); for women with no use of AIT, the numbers were 5,473 (median pr. women of 2) and 10,210 (median pr. women of 3). Figure S3 shows the distribution of the LDL-cholesterol measurements for women with and without use of AIT. 4692 out of 10,461 women (45%) had at least one post-breast cancer LDL-cholesterol measurement exposed to lipid-lowering therapy (44.3% for no use of AIT and 45.0% for use of AIT).

### Analysis of primary outcome

For 7919 women with use of AIT (> 99% had letrozole as first dispensed AIT), the mean pre-breast cancer LDL-cholesterol level was 2.94 mM and the mean post-breast cancer LDL-cholesterol level was 2.78 mM, corresponding to a mean change of − 0.16 mM (Table 2). For women with no use of AIT, the mean pre-breast cancer LDL-cholesterol level was 2.97 mM and the mean post-breast cancer LDL-cholesterol level was 2.82 mM, corresponding to a mean change of − 0.15 mM. The corresponding adjusted difference in LDL-cholesterol-change was − 0.03 mM (95% CI − 0.07 to 0.003) for women with use of AIT versus no use of AIT. We found similar estimates with stratification according to various subgroups including history of ischemic CVD and time since start of the time window for included post-breast cancer lipid measurements, and post hoc, we additionally subgrouped by age, use of LLD, history of hypertension, and history of diabetes (Table 3 and Table S3).

**Table 2:** Difference in LDL-cholesterol-change according to use of AIT in a nationwide cohort of postmenopausal women with early breast cancer, January 2009 to December 2020, Denmark

	Women	Pre-breast cancer LDL-cholesterol^a^	Post-breast cancer LDL-cholesterol		LDL-cholesterol-change	Difference in LDL-cholesterol-change	
	*n*	Mean (mmol/L)	*n*	Mean (mmol/L)	Mean (mmol/L)	Crude (mmol/L)	Adjusted^b^ (mmol/L)
No use of AIT	2542	2.97	10,210	2.82	− 0.15^c^	0 ref	0 ref
Use of AIT	7919	2.94	32,540	2.78	− 0.16^c^	− 0.02(− 0.05,0.01)	− 0.03(− 0.07,0.003)

AIT, aromatase inhibitor treatment; LDL, low density lipoprotein.

‘Use of AIT’ is defined as allocated and dispensed treatment with AIT, and ‘No use of AIT’ is defined as no allocated and no dispensed treatment with AIT; both as recorded in the clinical database of the Danish Breast Cancer Group (see Table S3 for further definitions).

^a^The pre-breast cancer LDL-cholesterol level is predicted using a linear effects model for all women with at least one LDL-cholesterol-measurement prior to breast cancer diagnosis (see Supplementary Methods for further details); the difference between the mean of the observed LDL-cholesterol-levels prior to breast cancer diagnosis and the predicted one was < 0.02 mmol/L.

^b^The model included, as fixed effects, use of AIT, demographic characteristics (age [2-year intervals], calendar period [1-year intervals], education, region of residence, disposable household income, and cohabitory status), tumor characteristics (laterality, size, histology and malignancy grade, lymph node involvement, human epidermal growth factor receptor 2-status), other anti-cancer treatments (type of surgery, chemotherapy, and radiotherapy), the interaction between laterality and radiotherapy, and the time of lipid-measurement; and a random intercept, to account for the inter-individual variability.

^c^The absolute change in pre- vs. post-breast cancer LDL-cholesterol is explained by age- and period-differences in LDL-cholesterol levels.

**Table 3:** Adjusted difference in LDL-cholesterol-change according to use of AIT in a nationwide cohort of postmenopausal women with early breast cancer, January 2009 to December 2020, Denmark, analyses with stratification according to subgroups

	Women (n, no use of AIT/use of AIT)	Adjusted difference in LDL-cholesterol change^a,b^
Main estimate	2542/7919	− 0.03 (− 0.07, 0.003)
Subgroup analyses		
*Time since start of the time window for included post-breast cancer lipid measurements*		
< *1 year*	1689/5468	− 0.02(− 0.07,0.02)
*1–2 years*	2014/6435	− 0.05(− 0.09,− 0.01)
*3–4 years*	1393/4342	− 0.03(− 0.07,0.02)
*History of ischemic CVD*^*c*^		
*No history of IHD or IS*	2123/6738	− 0.04(− 0.08,− 0.004)
*History of IHD or IS*	419/1181	0.02(-0.05,0.10)
*Age*^*c*^		
< *70 years*^*d*^	1704/4496	− 0.05(− 0.09,− 0.01)
≥ *70 years*^*d*^	838/3423	− 0.003(− 0.06,− 0.05)
*Lipid lowering drugs*^*c*^		
*No lipid lowering drugs*	1556/4875	− 0.04(− 0.09,− 0.0001)
*Lipid lowering drugs*	986/3044	− 0.02(− 0.08,0.03)
*History of hypertension*^*c*^		
*No history of hypertension*	1371/4086	− 0.05(− 0.09,− 0.001)
*History of hypertension*	1171/3833	− 0.02(− 0.07,0.03)
*History of diabetes*^*c*^		
*No history of diabetes*	2195/6806	− 0.03(− 0.07,0.004)
*History of diabetes*	347/1113	− 0.03(− 0.11,0.05)

AIT, aromatase inhibitor treatment; BMI, body mass index, CVD, cardiovascular disease; IHD, ischemic heart disease; IS, ischemic stroke.

‘Use of AIT’ is defined as allocated and dispensed treatment with AIT, and ‘No use of AIT’ is defined as no allocated and no dispensed treatment with AIT; both as recorded in the clinical database of the Danish Breast Cancer Group (see Table S3 for further definitions).

^a^The model included, as fixed effects, use of AIT, demographic characteristics (age [2-year intervals], calendar period [1-year intervals], education, region of residence, disposable household income, and cohabitory status), tumor characteristics (laterality, size, histology and malignancy grade, lymph node involvement, human epidermal growth factor receptor 2-status), other anti-cancer treatments (type of surgery, chemotherapy, and radiotherapy), the interaction between laterality and radiotherapy, and the time of lipid-measurement; and a random intercept, to account for the inter-individual variability.

^b^The *p*-values (testing for homogeneity) were as follows: time since start of the time window for included post-breast cancer lipid measurements 0.31, history of ischemic CVD 0.10, age at baseline 0.17, lipid lowering drugs 0.50, hypertension 0.35, and diabetes 0.95.

^c^Status for the subgrouped covariate is assessed at index date (defined as date of breast cancer diagnosis (surgery date or biopsy date if neoadjuvant treatment)).

^d^The cut-off for age was chosen based on the median (see Table 1)

We performed several sensitivity analyses for the primary outcome and found similar results. This included analyses applying alternative exposure definitions: comparing women allocated to AIT with women not allocated to AIT, updating use of AIT during the time window for included post-breast cancer lipid measurements, and comparing women with current or previous use of AIT with women with never use of AIT (Table S7). Furthermore, we conducted analyses ending the time window for included post-breast cancer lipid measurements at first use of tamoxifen, and at diagnosis of contra-lateral or recurrent breast cancer or other cancer; disregarding LDL-cholesterol measurements when the corresponding triglyceride value was above 4.5 mM; including only one post-breast cancer LDL-cholesterol measurement; excluding patients who received neoadjuvant treatment, and performing further adjustments, including a post hoc analysis with time dependent use of LLD (Table S7).

### Analysis of secondary outcomes

For the secondary outcomes HDL-cholesterol, total cholesterol and triglycerides the adjusted difference in change in lipid-level was − 0.01 mM (95% CI − 0.03 to 0.001), − 0.04 mM (95% CI − 0.09 to − 0.003), and 0.02 mM (95% CI − 0.01 to 0.05), respectively, for women with use of AIT versus no use of AIT (Table 4).

**Table 4:** Difference in change in secondary outcomes (HDL-cholesterol, total cholesterol and triglycerides) according to use of AIT in a nationwide cohort of postmenopausal women with early breast cancer, January 2009 to December 2020, Denmark

	Women	Pre-breast cancer-value^a^	Post-breast cancer-value		Change in secondary outcome	Difference in change in secondary outcome	
	*n*	Mean (mmol/L)	*n*	Mean (mmol/L)	Mean (mmol/L)	Crude (mmol/L)	Adjusted^b^ (mmol/L)
Secondary outcomes							
*HDL-cholesterol*							
No use of AIT	2542	1.71	10,423	1.68	-0.02	0 ref	0 ref
Use of AIT	7917	1.69	33,074	1.64	-0.05	-0.02(-0.03,-0.01)	-0.01(-0.03,0.001)
*Total cholesterol*							
No use of AIT	2541	5.32	10,565	5.19	-0.14	0 ref	0 ref
Use of AIT	7917	5.29	33,576	5.14	-0.16	-0.03(-0.06,0.01)	-0.04(-0.09,-0.003)
*Triglycerides*							
No use of AIT	2531	1.48	10,487	1.58	0.10	0 ref	0 ref
Use of AIT	7893	1.53	33,325	1.65	0.13	0.03(0.004,0.05)	0.02(-0.01,0.05)

AIT, aromatase inhibitor treatment.

Only women with a measured pre-breast cancer level of HDL-cholesterol, total cholesterol or triglycerides, respectively, and a measured post-breast cancer level of HDL-cholesterol, total cholesterol or triglycerides, were included in the correspondent analysis.

‘Use of AIT’ is defined as allocated and dispensed treatment with AIT, and ‘No use of AIT’ is defined as no allocated and no dispensed treatment with AIT; both as recorded in the clinical database of the Danish Breast Cancer Group (see Table S3 for further definitions).

^a^The pre-breast cancer LDL-cholesterol level is predicted using a linear effects model for all women with at least one LDL-cholesterol measurement prior to breast cancer diagnosis (see Supplementary Methods for further details); the difference between the mean of the observed LDL-cholesterol measurement prior to breast cancer diagnosis and the predicted one was < 0.02 mmol/L.

^b^The model included, as fixed effects, use of AIT, demographic characteristics (age [2-year intervals], calendar period [1-year intervals], education, region of residence, disposable household income, and cohabitory status), tumor characteristics (laterality, size, histology and malignancy grade, lymph node involvement, human epidermal growth factor receptor 2-status), other anti-cancer treatments (type of surgery, chemotherapy, and radiotherapy), the interaction between laterality and radiotherapy and the time of lipid-measurement; and a random intercept, to account for the inter-individual variability.

## Discussion

In this nationwide study, we found no association between use of AIT in women with postmenopausal breast cancer and change in LDL-cholesterol as compared with no use. The findings were adjusted for important confounders including indications for measurement of lipid-levels and remained at a similar level irrespective of time since start of follow-up, current vs. past use, and presence or absence of a history of ischemic CVD. For the secondary outcomes (change in total cholesterol, HDL-cholesterol and triglycerides), we found similar levels of association.

Despite several previous studies on this issue, uncertainty remains due to limited generalizability to everyday clinical practice. Data from a systematic review; however, do not show any definitive pattern of unfavorable changes of AIT on plasma-lipoproteins from baseline to follow-up assessments in patients with hormone receptor-positive early breast cancer; however, interpretability is limited by heterogeneity in the patient population, treatment sequence (i.e. initial AIT or a setting with switch between tamoxifen and AIT) and dissimilarities in data collection methods [3]. The review included 15 RCTs investigating AIT either as initial adjuvant therapy (three trials), switch therapy (seven trials) or in the extended adjuvant setting (two trials). However, among the three trials that investigated AIT as initial adjuvant therapy (i.e. corresponding to the setting of our study), only one had a placebo-based comparator group [24, 31]. This study was a small RCT (2 years of exemestane vs. placebo) that found a 6–9% drop in HDL-cholesterol levels for exemestane compared with 1–2% for placebo after 24 months treatment [31]; however, the drop was reverted 1 year after cessation of AIT, and no significant differences were observed for the remaining measurement time points [24]. In our study, first dispensed AIT was letrozole for most cohort members, limiting generalizability to other third-generation AITs; nevertheless, our findings are overall in line with this previous small RCT. To sum up, the results of our nationwide study which is based on rich information about breast cancer characteristics and adjustment variables, expands on existing evidence by providing substantial reassurance that AIT do not adversely affect lipid-levels in patients treated as part of contemporary everyday clinical practice including in patients with no previous tamoxifen treatment and according to previous history of ischemic CVD.

Strengths of the study include the nationwide design with assignment of treatment according to national guidelines minimizing selection bias, loss to follow-up, and maximizing power and generalizability to today’s treatment setting. Moreover, the study size allowed for stratification according to history of ischemic CVD and use of lipid-lowering therapy. The study also had limitations. First, our study is based on longitudinal lipid-level information from a national database that did not include information on indication for testing. However, it is unlikely that it has affected our conclusion as treatment assignment is based on known guideline factors that are included in the measurement probability weights in the statistical model, and AIT is not mentioned as an indication for monitoring lipid-levels in current Danish breast cancer guidelines [30] (see supplementary material for further discussion). Second, although both AIT and lipid-levels are related to mortality, this does not affect our conclusion as AIT is primarily related to cancer mortality and lipid-levels to cardiovascular death. Finally, we had access to information about a rich range of confounding factors; however, we did not adjust for alcohol use or hereditary lipid disorders. Results were similar when adjusted for BMI and smoking in the subset where this information was available. LDL-cholesterol levels are affected by LLD; however, adjustment for post-breast cancer treatment with LLD did not affect the results.

The 2022 cardio-oncology guidelines from the European Society of Cardiology recommend baseline cardiovascular risk assessment and estimation of 10-year fatal and non-fatal CVD risk before use of endocrine treatment for breast cancer (i.e. including lipid levels) in patients without pre-existing CVD and annual cardiovascular risk assessment is recommended during endocrine therapy in breast cancer patients with high 10-year risk of cardiovascular events [32]. Recent consensus recommendations from the European Society of Medical Oncology on management of cardiac disease in cancer recommend lipid panel prior to potential use of cardiotoxic agents, including AIT [33]. To put the magnitude of the differences observed in the present study into perspective, current guidelines for primary prevention of CVD in individuals < 70 years at very high risk, recommend an ultimate LDL-cholesterol goal of < 1.4 mmol/L and LDL-cholesterol reduction of > 50% from baseline [6]. A 1 mM reduction in LDL-cholesterol has been shown to reduce the annual rate of major vascular events by about a fifth [34] i.e. a 0.05 mM lowering in LDL-cholesterol corresponds to a 1% reduction in the annual rate of major vascular events and a 0.1 mM lowering to a 2% reduction. In this perspective, we do not consider the differences and their confidence limits observed in the present study clinically important.

To conclude, the results of the present study do not suggest that AIT adversely affects levels of LDL-cholesterol, nor HDL-cholesterol, triglycerides or total cholesterol. Breast cancer patients may receive a range of different potential cardiotoxic therapies simultaneously or in succession, and current guidelines reflect this in terms of recommended cardiovascular work-up and monitoring. Nevertheless, the results of this study do not indicate, that AIT per se is a concern in terms of a detrimental effect on plasma lipids.

## Supplementary Information

Below is the link to the electronic supplementary material.

Supplementary file1 (DOCX 415 KB)

## Declarations

### Approvals

The study was approved by Statens Serum Institut’s Data Protection and Information Security Department; under Danish law, neither informed consent nor ethics committee approval is required for strictly register-based studies.
