# Supplementary material for: Changes in LDL-cholesterol levels following aromatase inhibitor treatment in early postmenopausal breast cancer
Source: Eur J Epidemiol. 2025 May 21;40(8):937–46. doi: 10.1007/s10654-025-01228-7 (PMC12374885; doi:10.1007/s10654-025-01228-7)
Supplement: Supplementary file 1 — Supplementary file1 (DOCX 415 KB) [file 10654_2025_1228_MOESM1_ESM.docx]

**Supplementary material**

**Manuscript title**

Changes in LDL-cholesterol levels following aromatase inhibitor treatment in early postmenopausal breast cancer

**Authors**

Marie Lund MD PhD, Giulia Corn MSc PhD, Maj-Britt Jensen MSc, Tonny Petersen MD PhD, Kim Dalhoff MD DMSc, Bent Ejlertsen MD DMSc, Lars Køber MD DMSc, Jan Wohlfahrt DMSc, Mads Melbye MD DMSc

[Table S1. Reasons for no inclusion in a treatment protocol in the clinical database of the Danish Breast Cancer Group (DBCG-database) in a nationwide cohort of postmenopausal women with early breast cancer, January 2009 to December 2020, Denmark 2](#_Toc196755159)

[Figure S1. Criteria for being assigned to the low- and high-risk prognostic groups, respectively, since 2009, according to the Danish Breast Cancer Cooperative Group 3](#_Toc196755160)

[Table S2. Overview of Data Sources 4](#_Toc196755161)

[Table S3. Definitions of inclusion and exclusion criteria, follow-up, exposure, outcome and covariates 5](#_Toc196755162)

[Figure S2. Illustration of study design 12](#_Toc196755163)

[Supplementary Methods 13](#_Toc196755164)

[Table S4. Covariates included in the statistical models 15](#_Toc196755165)

[Table S5. Distribution of additional covariates according to use of AIT^a^ 17](#_Toc196755166)

[Figure S3. Distribution of the included pre-breast cancer and post-breast cancer LDL-cholesterol measurements in the two time windows in a nationwide cohort of postmenopausal women with early breast cancer with and without use of AIT, January 2009 to December 2020, Denmark 19](#_Toc196755167)

[Table S6. Difference in LDL-cholesterol-change according to use of AIT^a^ in a nationwide cohort of postmenopausal women with early breast cancer, January 2009 to December 2020, Denmark, analyses with stratification according to subgroups^b^ 20](#_Toc196755168)

[Table S7. Difference in LDL-cholesterol-change according to use of AIT^a^ in a nationwide cohort of postmenopausal women with early breast cancer, January 2009 to December 2020, Denmark, sensitivity analyses 22](#_Toc196755169)

[Potential bias in relation to use of the register-based lipid-measurements 25](#_Toc196755170)

[Reference list 26](#_Toc196755171)

# **Table S1. Reasons for no inclusion in a treatment protocol in the clinical database of the Danish Breast Cancer Group (DBCG-database) in a nationwide cohort of postmenopausal women with early breast cancer, January 2009 to December 2020, Denmark**

| **Reason^a^** | **N** | **%** |
| --- | --- | --- |
| Metastatic or locally advanced breast cancer | 2054 | 38.5 |
| Bilateral breast cancer | 125 | 2.3 |
| Surgery not according to guidelines^b^ | 2672 | 50.1 |
| Miscellaneous (contraindication, death within 4 weeks after surgery or reason designated as other) | 43 | 0.8 |
| No reason reported (none of the above) and missing information necessary for inclusion in protocol | 273 | 5.1 |
| No reason reported and complete information necessary for inclusion in protocol | 169 | 3.2 |
| ^a^ In the DBCG-database more than one reason for no inclusion in a treatment protocol can be reported; in the above table, reason for no inclusion in a treatment protocol is for simplicity listed hierarchically from top to bottom in the table, i.e. women are in this table only listed according to one specific reason.  ^b^ i.e. no breast-surgery or no sentinel lymph node dissection/axillary lymph node dissection. | | |

# **Figure S1. Criteria for being assigned to the low- and high-risk prognostic groups, respectively, since 2009, according to the Danish Breast Cancer Cooperative Group**


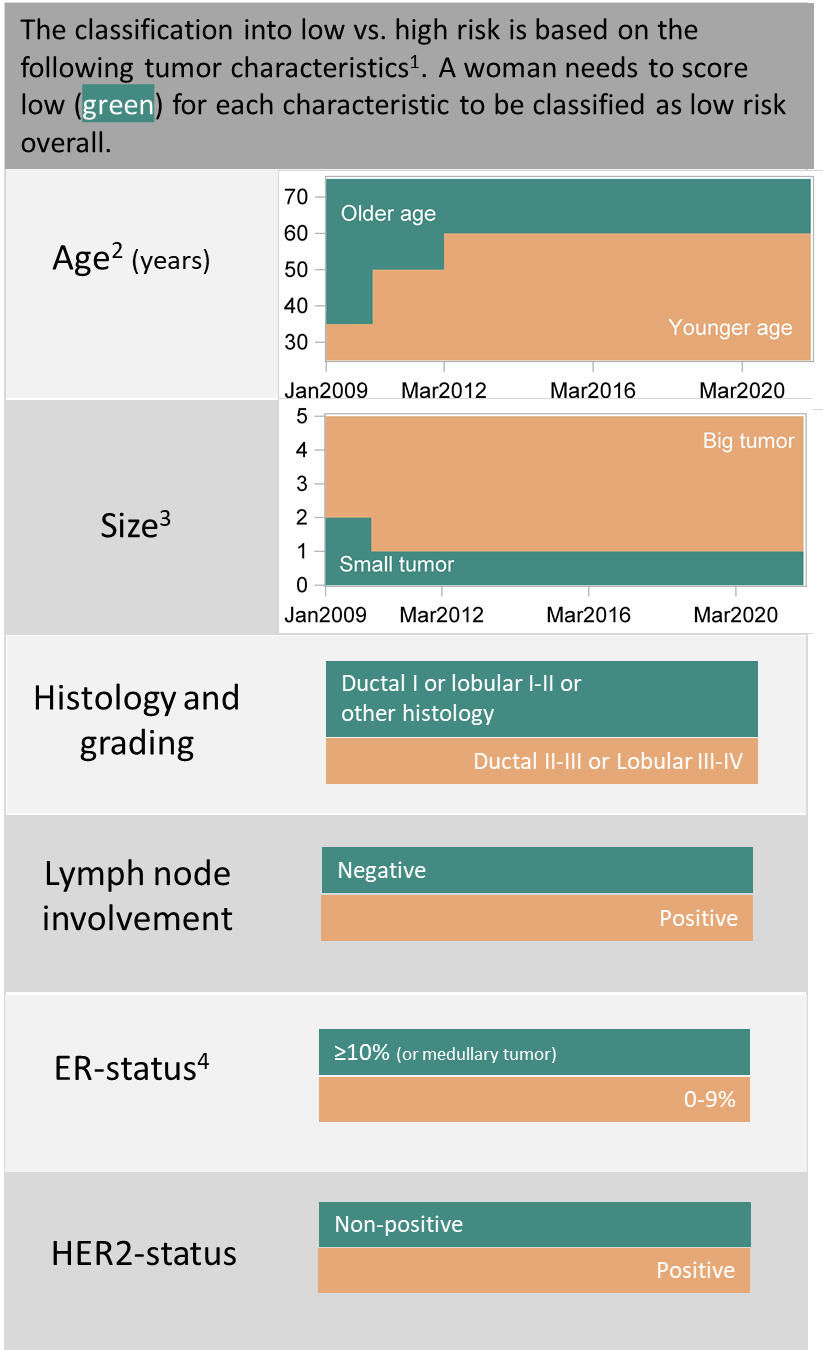


Abbreviations: ER (estrogen receptor), HER2 (human epidermal growth factor receptor 2)

^1^ Topoisomerase 2 alpha-amplification has been included as criterium for belonging to the high-risk group if abnormal from start of study period until July 2013 ^1^ i.e. not for the entire study period and is therefore not included in the figure above.

^2^ The age cut-off for belonging to the high-risk group was < 35 years from the start of study period to March 2010, < 50 years until March 2012 and < 60 years thereafter ^1^.

^3^ The cut-off for tumor size to belong in the high-risk group was > 2 cm from the start of study period to March 2010 and > 1 cm thereafter.

^4^ Before March 2010, evaluation of hormone receptor status included an overall assessment of both estrogen and progesterone receptor status (with classification based on the most positive among both), whereas only estrogen receptor status but not progesterone receptor status was used thereafter ^2^.

| **Table S2. Overview of Data Sources** | |
| --- | --- |
| **Data source (period covered)** | **Content** |
| The Danish Central Person Register (CPR, 1968 onwards) ^3^ | Demographic information including vital status, place of birth, gender, co-habitory status and kinship for all individuals resident in Denmark. |
| Clinical database of the Danish Breast Cancer Cooperative Group (DBCG, 1978 onwards) ^4^ | Information on incident breast cancer^a^ since 1978 incl. diagnostic and prognostic information (e.g. HER2-status, ER-status, tumor characteristics incl. histology, malignancy grade and number of positive lymph nodes), therapeutic information (chemotherapy, endocrine therapy, HER2-directed therapy, radiation therapy, surgery), BMI and follow-up information on breast cancer recurrence and contralateral breast cancer. |
| National Patient Register (NPR, inpatients since 1977, outpatients since 1995) ^5^ | Information on diagnoses and procedures for all individual patient contacts at Danish hospitals. |
| The Danish National Prescription Registry (DNPR, 1995 onwards) ^6^ | Information on filled prescriptions at Danish pharmacies including e.g. specific ATC-code, strength and pack size. |
| The Register of Laboratory Results for Research (LAB, 2008 onwards) ^7,8^ | Information on individual-level results of laboratory tests performed at Danish clinical biochemical laboratories with continuous inclusion of data from the various regions since 2008. |
| Statistics Denmark (period covered depends on type of information registered) ^9–11^ | Information on various demographic information incl. socioeconomic data such as educational attainment (since 1980), disposable household income (since 1987 and co-habitory status (since 1985). |
| Danish Anaesthesia Database (DAD, 2004 onwards) ^12^ | Information on BMI and smoking for all patients who have undergone surgery. |
| The Pathology Register (PAT, 1970 onwards) ^13^ | Information about pathology data from all departments of pathology in Denmark (mandatory reporting since 1997) with information recorded using the Danish SNOMED coding system for pathology diagnoses. |
| The Cancer Register (CAR, 1943 onwards) ^14^ | Tumor characteristics for incident cancers in the Danish population. |
| Abbreviations: ATC (anatomic therapeutic chemical classification), BMI (body mass index), ER (estrogen receptor), HER2 (human epidermal growth factor receptor 2). ^a^ For the construction of the study cohort, we also used information about women registered with breast cancer in the DBCG Quality Database for Breast Cancer (Danish Clinical Quality Program – National Clinical Registries (RKKP)) ^15^. | |

| **Table S3. Definitions of inclusion and exclusion criteria, follow-up, exposure, outcome and covariates** | | | |
| --- | --- | --- | --- |
| **Variable** | **Definition** | **Data source** | **Percentage of missingness and how it was handled** |
| **Inclusion criteria** | | | |
| Female breast cancer | Included in the DBCG-database | DBCG | - |
| Postmenopausal |  | DBCG | - |
| Valid CPR-nr | According to the Central Person Register | CPR | - |
| Period of inclusion (01/2009-12/2020) | Defined based on the index date | DBCG | - |
| **Exclusion criteria** | | | |
| Other primary cancer | Other primary cancer excluding non-melanoma skin cancer ^16^: ICD10: CXX.X\(C44+C46.0 + C50)+D09.0+D41.4+D32-33+D42-43  ICD7: 140-204\(191+170) | CAR | - |
|  | Reason for no protocol allocation stated as previous malignant disease in the DBCG-database | DBCG |  |
| Not in protocol |  | DBCG | - |
| No address in Denmark two years prior to index date | Defined by use of address information from the Central Person Register | CPR | - |
| Recurrent, or contra-lateral breast cancer | Recurrent, or contralateral breast cancer as recorded in the DBCG-database | DBCG | - |
| Emigration |  | CPR | - |
| Date of death |  | CPR | - |
| **Time windows for included lipid measurements** | | | |
| Index date | Date of breast cancer diagnosis defined as surgery date or biopsy date if neoadjuvant treatment | DBCG | - |
| End of chemotherapy | Latest registered date with dispensed chemotherapy | DBCG | - |
| Time window for included pre-breast cancer lipid measurements | From 2 years prior to index date to index date |  | - |
| Start of time window for included post-breast cancer lipid measurements | 3 months after index date or end of chemotherapy whichever came last | DBCG | - |
| End of time window for included post-breast cancer lipid measurements | Emigration, death, end of study period, or 5 years after start date of the time window whichever came first. | CPR | - |
| **Exposure** | | | |
| Allocation to AIT | Allocated or not allocated to endocrine treatment (since the cohort consists of postmenopausal women, we assumed this to be AIT). | DBCG | - |
| Dispensed AIT | ‘Dispensed AIT’ includes women with ≥1 registration of dispensed AIT.  ‘No dispensed AIT’ includes women with a registration of no endocrine treatment, and women for whom no registration of endocrine treatment is available. Of note, women to whom tamoxifen was dispensed before the start of the time window for included breast cancer lipid measurements were excluded from the study. | DBCG | - |
| Use of AIT | Allocated to and dispensed AIT | DBCG | - |
| No use of AIT | No allocation to and no dispensed AIT | DBCG | - |
| Analyses with exposure updated during the time window for included post-breast cancer lipid-levels | Current/past/never use of AIT defined based on dispensed AIT. A woman is considered current user of AIT until 12 months after the latest dispensed AIT, after which the woman is considered past user. Never use includes women with no allocation to and no dispensed AIT (i.e. similar definition as the definition ‘No use of AIT above, but time-updated). In particular, for women not allocated to AIT, who dispensed AIT for the first time during the time window for included post-breast cancer lipid-measurements, the lipid measurements before the date of first dispensed AIT are considered as among never users, while measurements after that date are excluded. | DBCG | - |
| **Lipid levels** | | | |
| LDL-cholesterol | NPU codes: NPU01568, NPU10171, or DNK35308 | LAB | - |
| HDL-cholesterol | NPU codes: NPU10157 or NPU01567 | LAB | - |
| Total cholesterol | NPU codes: NPU01566 or NPU18412 | LAB | - |
| Triglycerides | NPU codes: NPU04094 or NPU03620 | LAB | - |
| **Adjustment variables** | | | |
| Calendar period | Year of index date | DBCG | - |
| Age | Age at index date | CPR | - |
| Region of residence | Region of residence at index date | CPR | - |
| Education | <10y, 10-12y, 13-15y, >=15y | DST | n=162 (1.5%) imputed as “10-12 years” |
| Disposable household income | 1^st^ to 4^th^ quartile | DST | - |
| Co-habitation status | Single (= no) or cohabitant (= yes) | DST | - |
| **Other oncological treatment^a^** | | | |
| Chemotherapy | Registration of treatment with chemotherapy grouped as chemotherapy yes/no | DBCG | n=287 (2.7%) imputed according to treatment protocol |
| Neoadjuvant treatment | Registration as having received surgery after neoadjuvant treatment | DBCG | - |
| Radiotherapy | Allocation to radiotherapy (yes/no) | DBCG | - |
| Type of surgery | Mastectomy or breast conserving surgery | DBCG | n=28 (0.3%) imputed as “breast conserving surgery” |
| **Tumor characteristics** | | | |
| Laterality | Left or right | DBCG | n<5 (0.0%) imputed as “left” |
| Tumor size | Grouped to reflect the criteria used for treatment protocol allocation: ≤1 cm, >1-2cm, >2-5 cm, >5 cm | DBCG | n=44 (0.4%) imputed as “≤1 cm” for women in the low-risk group, and “>1-2 cm” for women in the high-risk group |
| Lymph node involvement | Negative, 1-3 positive lymph nodes, 4-9 positive lymph nodes, ≥10 positive lymph nodes | DBCG | n=55 (0.5%) imputed as “no/negative” |
| Histology and grading | Ductal*,* lobular or other type. Ductal and lobular tumors were graded during the study period and hence they were further categorized according to their malignancy grade (1, 2, or 3) | DBCG | n=83 (0.8%) imputed as “ductal grade 1” for women in the low-risk group, and “ductal grade 2” for women in the high-risk group |
| HER2-status | Positive or negative/normal | DBCG | N=121 (1.2%) imputed as “negative/normal” |
| **Indications for measurement of lipid levels in primary practice** (A/B diagnosis, in/out patient, look-back period= ∞, unless otherwise specified) | | | |
| Any CVD | ICD10: I01, I02.0, I05-I99, R57.0, R96.0, R96.1.  SKS (inpatient only): KFNA-G, KFNH20, KFPE00, KFPE10, KFPE20, KFPE96, KFPF00, KFPF10, KFPF20, KFPF96, KFPG, BFCA0, BFCA6, BFCB0, BFCB6. | NPR | - |
| Ischemic heart disease (incl. revascularization procedures) | ICD10: I20-25 or SKS (inpatient only): KFNA-G, KFNH20 | NPR | - |
| Stroke | ICD10: I63-64 | NPR | - |
| Heart failure | ICD10: I11.0, I13.0, I13.2, I42.0, I42.6-9, I50.0, I50.1, I50.9, J81 | NPR | - |
| Essential hypertension | ICD10: I10-I15 OR combination treatment with at least two of the following classes of antihypertensive drugs within 1 year prior to index date:  • thiazides or thiazide-like diuretics (ATC: C03A, C03B, C03EA01, C09BA, C09DA, C09DX01, C09XA52)  • selective calcium channel blockers (ATC: C07F, C08CA, C09BB, C09DB, C09DX01)  • Renin-angiotensin system inhibitors (ATC: C09) | NPR, DNPR | - |
| Familial disposition to premature ASCVD ^17^ | At least one first degree relative (according to kinship-information in the CPR) with one of the following registrations (for women < 60y and men < 55y):  Ischemic heart disease (ICD10: I20-I25, SKS (procedure codes: inpatients only): KFNA-E, KFNH20, KFNG, KFNF), stroke and TIA (ICD10: I61, I63-I64, G45) and peripheral arterial disease (ICD10: I70, I712, I714, I716, I719, I72, I738, I739, I74, I77) | CPR, NPR | - |
| Moderate/severe impaired renal dysfunction | LAB: eGFR < 60 mL/min/1,73 m2 (last two measurements prior to index date below the threshold; maximum look-back 2 years prior to index date). DNK35131 or DNK35302 used for eGFR and NPU04998 or NPU18016 used for creatinine from which the eGFR is calculated using the CKD-EPI formula without race correction.  LPR:  ICD10: N18 OR (inspired by ^18^):   1. The patient has been in dialysis (SKS code BJFD) at least 12 times (for at least one period) of 90–97 days after 1 January 2000, in which either the distance between the first and last dialysis is 90–97 days, OR 2. at least one dialysis was reported as chronic peritoneal dialysis (SKS codes: BJFD21, BJFD22, BJFD23, BJFD24, BJFD25 or BJFD27). | LAB, NPR | - |
| Diabetes (type 1 diabetes mellitus or type 2 diabetes mellitus) | ICD10: E10-E14, ATC: at least two prescriptions for A10; EXCLUDING: All diagnosis and medicine contacts from and 1 year after first diagnosed with O24.4 (pregnancy diabetes) AND/OR if women are diagnosed with E282 (PCOS) OR have been treated with medicine for polycystic ovary syndrome as defined by ATC: A10BA02 in combination with either ATC: G03GB02 or G03HB (definition inspired by ^18^). | NPR, DNPR | - |
| Obstructive sleep apnea | ICD10: G47.32, Z99.8A | NPR | - |
| COPD | ICD10: J44 | NPR | - |
| Smoking | The non-missing value closest to the index-date. Values from 2 years before to 6 months after index date are considered.  Yes: includes the answers “Yes” “Smoker or cessated within 8 weeks”  No: includes “never smoker”, “no-smoker”, and ”former smoker” | DAD | N=4100 (39.2%) in the analysis where this variable was used, complete case analysis was applied |
| BMI | The non-missing value closest to the index-date. Values from 2 years before to 6 months after index date are considered. | DAD, DBCG | N=2217 (21.2%) in the analysis where this variable was used, complete case analysis was applied |
| Pancreatitis | ICD10: K85, K860, K861, B252, B263 |  | - |
| Use of thiazides | ≥2 prescriptions with the following ATC codes within 1 year prior to index date  ATC: C03A, C03B, C03EA01, C09BA, C09DA, C09DX01, C09XA52 | LAB | - |
| Use of beta-blockers | ≥2 prescriptions with the following ATC codes within 1 year prior to index date  ATC: C07 excl. C07AB12 | DNPR | - |
| Use of oral estrogen replacement | ≥2 prescriptions with the following ATC codes within 1 year prior to index date ATC: G03CA, G03F | DNPR | - |
| Pre-breast cancer lipid lowering drugs | ≥2 prescriptions with the following ATC codes within 1 year prior to index date ATC: C10.  Definition of intensity (based on 2019 ACC/AHA guideline for lipid-lowering treatment ^19^):   - Low  simvastatin 10 mg (C10AA01),  fluvastatin 20-40 mg (C10AA03),  lovastatin 20 mg (C10AA02) or  pravastatin 20 (C10AA04) - Moderate:  simvastatin 20-80 mg (C10AA01),  fluvastatin 80 mg (C10AA03),  lovastatin 40 mg (C10AA02),  pravastatin 40 (C10AA04),  atorvastatin 10-20 mg (C10AA05) or rosuvastatin 5-10 mg (C10AA07) - High:  atorvastatin 40-80 mg (C10AA05) or rosuvastatin 20-40 mg (C10AA07) - Statin in combination:  C10AA and (C10AB, C10AC, C10AD or C10AX) or C10BA   Lipid lowering drugs other than statins: C10A excl. C10AA | DNPR | - |
| Time dependent use of lipid lowering drugs during the time window for included post-breast cancer lipid measurements | ≥1 prescription with the following ATC codes within 100 days preceding the sampling date for LDL-cholesterol, ATC: C10. Intensity is defined as described above. |  | - |
| Use of antipsychotics | ≥2 prescriptions with following ATC codes within 1 year prior to index date ATC: N05A | DNPR | - |
| Hospitalized | Yes/no  Inpatient contact:  In LPR2: *C_pattype=0,1* In LPR3: *kontakttype=ALCA00* and over-night stay | NPR | - |
| Out-patient contacts | Number of days with a non-over-night hospital contact   - In LPR3 kontakttype=ALCA00   In LPR2 no restriction on c_pattype | NPR | - |
| Medication use | Number of different ATCs-codes filled | DNPR | - |
| **Variables included in additional/sensitivity analyses** | | | |
| Use of tamoxifen | At least one registration with dispensed treatment with tamoxifen. | DBCG | - |
| Recurrent, metastatic, or contra-lateral breast cancer, or other cancer | Defined to censor at recurrent, contralateral breast cancer or other malignant disease (as recorded in the DBCG-database) | DBCG | - |
|  | Defined to censor at other cancer than breast cancer ^16^: ICD10: CXX.X\(C44+C46.0 + C50)+D09.0+D41.4+D32-33+D42-43 | CAR | - |
|  | Defined to censor at other cancer or recurrent breast cancer: ICD10: C76-C80  From 1 year after start of follow-up (rationale: to minimize mix-up with the primary breast cancer); adapted from ^20^. | NPR | - |
|  | Defined to censor at recurrent breast cancer or other cancer (SNOMED CODES):   1. T codes related to the breast  (T02400, T02401, T02424, T0242K, T0242L, T02430, T0243A, T0243B, T03400, T04000, T04001, T04002, T04010, T04020, T04030, T04100, T0410A, T0410B, T04200, T04202, T0420A, T0420B, T0420E, T0420F, T04280, T0428A, T0428B, T04400, T04441, T04442, T04600, T04800, T08350, T08352, T08353, T08354, T0835A, T0835B, T0835C, T0835D, T08710, T08711, T08712, T0871A, T0871B, T0871C, T08720, T08721, T08722, T09420, T0Y400, T0Y40A, T0Y40B, TY8101, TY8102, T08200, T08201, T08202, T0820A, T0820B, T0820C, T08210, T08211, T08212, T08220, T08221, T08222, T08280, T08281, T08282, TY062A, TY062B, TY123A, TY123B, TY1960, TY2960, TY8100) **and** M codes starting with M8 or M9 and having 3,4,6,7,9 in the fifth position (e.g. M8xxx4) **or** 2. M codes starting with M8 or M9 and having 6 or 9 in the fifth position   From 1 year after start of follow-up (rationale: to minimize mix-up with the primary breast cancer); adapted from ^20^. | PAT | - |
|  | Defined to censor at other cancer or recurrent breast cancer:  SKS: BWG, BWHA, KHAC, KHAB, KGAE16 (cancer-directed treatment codes).  From 1 year after start of follow-up (rationale: to minimize mix-up with the primary breast cancer); adapted from ^20^. | NPR | - |
|  | Defined to censor at recurrent breast cancer:  ICD10: DC509X (local breast cancer recurrence) or surgery code KHAF (an SKS code specific for recurrence surgery). From 1 year after start of follow-up (rationale: to minimize mix-up with the primary breast cancer); adapted from ^20^. | NPR | - |
| Abbreviations: AIT aromatase inhibitor treatment, ASCVD atheroschlerotic cardiovascular disease, BMI body mass index, COPD chronic obstructive pulmonary disorder, CPR Central Person Register, CVD cardiovascular disease, DAD The Danish Anaesthesia Database, DBCG The clinical database of the Danish Breast Cancer Cooperative Group, DNPR Danish National Prescription Register, DST Statistics Denmark, HER2 (human epidermal growth factor receptor 2), HDL high-density lipoprotein, ICD International Classification of Diseases, LAB The Register of Laboratory Results for Research, NPR National Patient Register, LDL low-density lipoprotein, PAT The Pathology Register, SKS Danish version of the Nordic Medico-Statistical Committee Classification of Surgical Procedures.  ^a^ We adjusted for three other oncological treatment variables: chemotherapy, radiation therapy and type of surgery. Radiation therapy was defined by status allocated whereas chemotherapy and type of surgery were defined using registered treatment as these variables were used to define start of the time-window for the included post-breast cancer lipid measurements. | | | |

# **Figure S2. Illustration of study design**


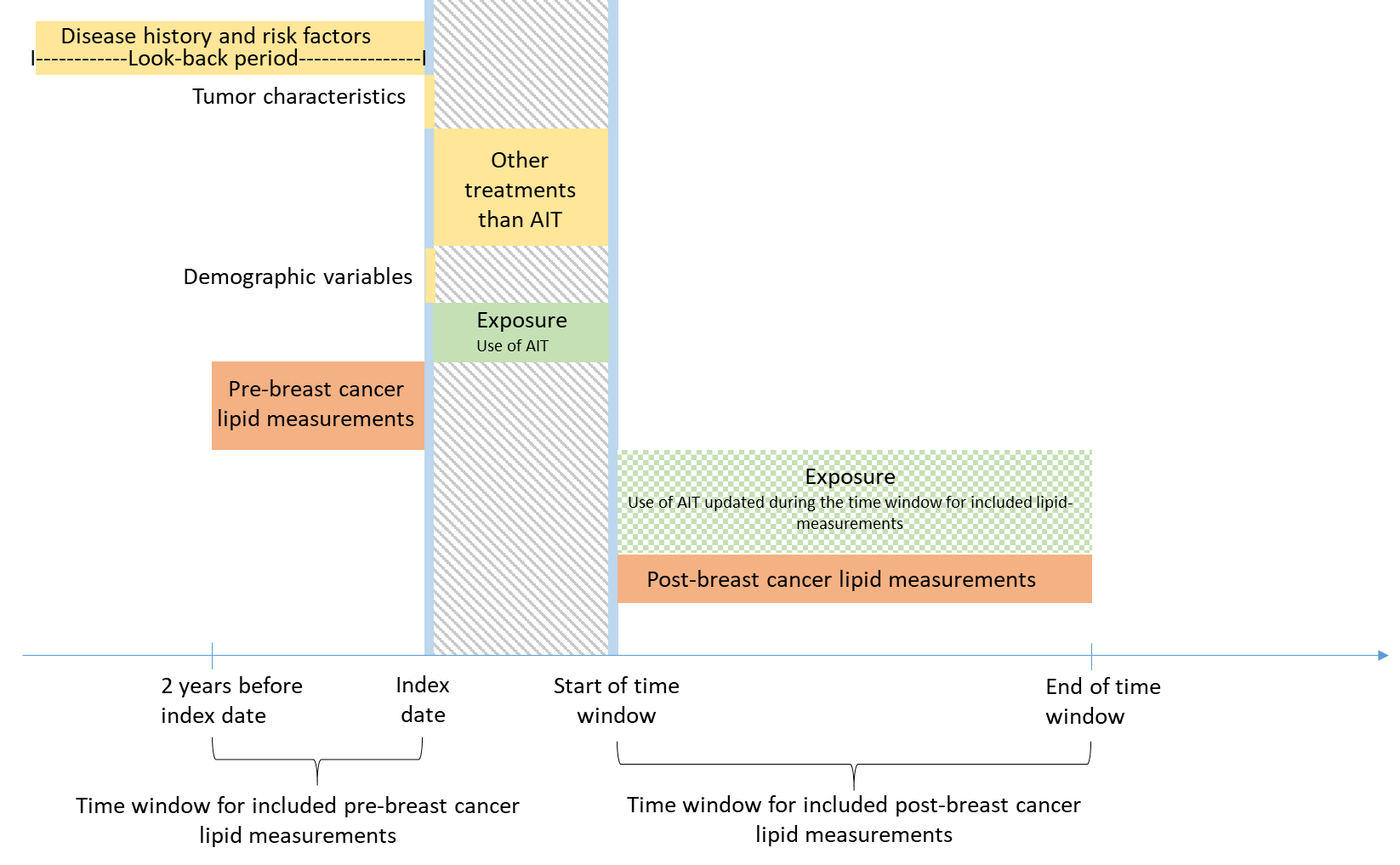


Abbreviations: AIT aromatase inhibitor treatment

Below is given a summary for each of the headings in the figure. Details of inclusion and exclusion criteria, exposure, outcome and covariates are given in Table S3.

- **Index date:** date of breast cancer diagnosis defined as surgery date or biopsy date if neoadjuvant treatment.
- **Time window for included pre-breast cancer lipid measurements:** Lipid measurements were ascertained 2 years prior to breast cancer diagnosis.
- **Time window for included post-breast cancer lipid measurements:** From 3 months after surgery for breast cancer or end of chemotherapy, whichever came last, and until date of emigration, death December 31, 2021 (end of study period), or 5 years after the start date of the time window.
- **Disease history and risk factors:** For specific look-back period for the individual covariates, see Table S3.
- **Other treatments than AIT:** Ascertained in the period from the index date to start of the time window for included post-breast cancer lipid measurements.
- **Demographic variables and tumor characteristics:** Determined at the index date.
- **Exposure:** The main exposure was allocated and dispensed endocrine treatment (termed ‘Use of AIT’) which was compared with no allocated and no dispensed AIT (termed ‘No use of AIT’). As additional secondary exposures, we compared current and past AIT use and duration of treatment with never dispensed AIT (no allocation to and no dispensed AIT).

# **Supplementary Methods**

To estimate the difference in change in lipid levels before and after breast cancer diagnosis according to use of AIT, we used a linear mixed-effects model with the change between the observed post-breast cancer lipid level (i.e. the primary outcome, low-density lipoprotein (LDL)-cholesterol, and the secondary outcomes, high-density lipoprotein cholesterol, triglycerides and total cholesterol, respectively) and the predicted pre-breast cancer lipid level as outcome, weighted for the probability of having a post-breast cancer LDL-cholesterol measurement. The predicted pre-breast cancer lipid level and the probability of having LDL-cholesterol measurements are estimated using three help models, which are described below in further detail in the order in which they were used (for individual covariates and interaction terms included in the models, see Table S4).

**Model of the probability of having a pre-breast cancer LDL-cholesterol measurement - model 1**

Among all women eligible for inclusion in the study (n=28,573), we estimated the probability of having at least one pre-breast cancer LDL-cholesterol measurement using logistic regression. The covariates included use of AIT, demographic variables (age, calendar period, region of residence, educational level, disposable household income, and cohabitation), tumor characteristics (tumor size, lymph node involvement, histology and grading, and HER2-status), indications associated with the measurement of lipid levels (pre-breast cancer use of lipid-lowering drugs, any cardiovascular disease, ischemic heart disease, stroke, heart failure, essential hypertension, familial disposition to premature ASCVD, impaired renal function, diabetes, obstructive sleep apnea, chronic obstructive pulmonary disease, smoking, BMI, pancreatitis, use of thiazides, use of beta-blockers, use of oral estrogen replacement, and use of antipsychotics), and contacts with health care (hospitalization, number of out-patient contacts, and medication use). Covariates presenting after time of breast cancer diagnosis, e.g. chemotherapy, were not included in model 1. These predicted probabilities were used to calculate the weight included in model 2 and 4 and were included as predictors in model 3.

**Model of the pre-breast cancer lipid level - model 2**

For all women with at least one pre-breast cancer LDL-cholesterol measurement (n=12,012), we predicted the individual pre-breast cancer lipid level using a linear mixed-effect model. Each woman contributed with a number of observations equal to the number of pre-breast cancer lipid level measurements. The model included the pre-breast cancer lipid level as outcome, one random intercept, to account for the inter-individual variability, and several fixed effects. The fixed effects included use of AIT, demographic variables (age, calendar period, region of residence, educational level, disposable household income, and cohabitation), tumor characteristics (tumor size, lymph node involvement, histology and grading, and HER2-status), and medical conditions associated with the measuring of lipid levels (pre-breast cancer use of lipid-lowering drugs, any cardiovascular disease, ischemic heart disease, stroke, heart failure, essential hypertension, familial distribution to premature ASCVD, impaired renal function, diabetes, obstructive sleep apnea, chronic obstructive pulmonary disease, smoking, BMI, pancreatitis, use of thiazides, use of beta-blockers, use of oral estrogen replacement, and use of antipsychotics). Covariates presenting after time of breast cancer diagnosis, e.g. chemotherapy, were not included in model 2. Each observation was weighted for the inverse of the probability of having a pre-breast cancer LDL-cholesterol measurement obtained from model 1.

**Model of the probability of having a post-breast cancer LDL-cholesterol measurement - model 3**

Among women with at least one pre-breast cancer LDL-cholesterol measurement (n=12,012), we estimated the probability of having at least one post-breast cancer LDL-cholesterol measurement using logistic regression. The covariates included demographic variables (age, calendar period, region of residence, educational level, household disposable income, and cohabitation), tumor characteristics (laterality, tumor size, lymph node involvement, histology and grading, and HER2-status), other oncological treatment (chemotherapy, radiotherapy, and type of surgery), the interaction between laterality and radiotherapy, indications associated with the measurement of lipid levels (pre-breast cancer use of lipid-lowering drugs, any cardiovascular disease, ischemic heart disease, stroke, heart failure, essential hypertension, familial disposition to premature ASCVD, impaired renal function, diabetes, obstructive sleep apnea, chronic obstructive pulmonary disease, smoking, BMI, pancreatitis, use of thiazides, use of beta-blockers, use of oral estrogen replacement, and use of antipsychotics), contacts with health care (hospitalization, number of out-patient contacts, and medication use) and information about the pre-breast cancer LDL-cholesterol level (probability of having a pre-breast cancer LDL-cholesterol level measured and the predicted LDL-cholesterol level). By multiplying the probabilities obtained from model 1, i.e. the probability of having a pre-breast cancer LDL-cholesterol measurement, and model 3, i.e. the probability of having a post-breast cancer LDL-cholesterol measurement given that a pre-breast cancer LDL-cholesterol measurement was registered, we obtained the probability of having a post-breast cancer LDL-cholesterol measurement.

**Model of the change in lipid levels - model 4 (already described in the main text)**

For all women with at least one pre-breast cancer LDL-cholesterol measurement and one post-breast cancer LDL-cholesterol measurement (n=10,461), we modelled the pre- vs. post-breast cancer change in lipid levels using a linear mixed-effects model. The outcome was the change between the observed post-breast cancer lipid level and the predicted pre-breast cancer lipid level. The model included one random intercept, to account for the inter-individual variability, and several fixed effects, including demographic variables (age, calendar period, region of residence, educational level, disposable household income, and cohabitation), tumor characteristics (laterality, tumor size, lymph node involvement, histology and grading, and HER2-status), other oncological treatment (chemotherapy, radiotherapy, and type of surgery), the interaction between laterality and radiotherapy, the time of the lipid measurement and use of AIT as the estimate of interest. Each woman contributed with a number of observations equal to the number of available post-breast cancer lipid measurements and all observations from the same women were weighted for the inverse of the probability of having a post-breast cancer LDL-cholesterol measurement (as estimated from model 3).

# **Table S4. Covariates included in the statistical models**

|  | **Pre-breast cancer models** | | **Post-breast cancer models** | |
| --- | --- | --- | --- | --- |
|  | Model 1: Predictive measure-ment model | Model 2: Predictive LDL-cholesterol model | Model 3: Predictive measure-ment model | **Model 4:**  **Main model (pre- vs. post-breast cancer change)** |
| **Exposure: use of AIT** | x | x |  | x |
| **Demographical variables (at index date)** |  |  |  |  |
| Calendar period (2009-11, 1-year intervals thereafter) | x | x | x | x |
| Age (<45y, 1-year intervals, ≥90y) | x |  | x |  |
| Age (<45y, 2-year intervals, ≥90y) |  | x |  | x |
| Region of residence | x | x | x | x |
| Education (4-level) | x | x | x | x |
| Household disposable income (4 quartiles) | x | x | x | x |
| Cohabitation (yes/no) | x | x | x | x |
| Period*region | x |  | x |  |
| Age (≤65y/>65y)*demographic variables | x |  | x |  |
| **Tumor characteristics** |  |  |  |  |
| Tumor size (<1, 1-2.0, 2.1-5.0, >5.0 cm) | x | x | x | x |
| Lymph node involvement (yes/no) | x | x | x | x |
| Histology and grading  (ductal grade I-III, lobular grade I- III, other histologies) | x | x | x | x |
| HER2-status (positive/negative) | x | x | x | x |
| **Other oncological treatment**  (not already accounted for in tumor characteristics) |  |  |  |  |
| Chemotherapy (yes/no) |  |  | x | x |
| Radiotherapy*laterality (yes/no*left/right) |  |  | x | x |
| Radiotherapy (yes/no) |  |  | x | x |
| Laterality (left/right) |  |  | x | x |
| Type of surgery (mastectomy/breast conserving surgery) |  |  | x | x |
| **Time of the lipid measurement**  **(months from the start of the time window for included post-breast cancer lipid measurements to actual measurement)** |  |  |  | x |
| **Indications for measurement of lipid levels in primary practice** | | | | |
| **Pre-breast cancer lipid lowering drugs** (none, low intensity statin, moderate intensity statin, high intensity statin, statin in combination with other lipid lowering drugs, other lipid lowering drugs) | x | x | x | (x) |
| **Cardiovascular variables (medical history)** |  |  |  |  |
| Any cardiovascular disease | x | x | x | (x) |
| Ischemic heart disease | x | x | x | (x) |
| Stroke | x | x | x | (x) |
| Heart failure | x | x | x | (x) |
| Essential hypertension | x | x | x | (x) |
| Familial disposition to premature ASCVD | x | x | x | (x) |
| Moderate/severely impaired renal function | x | x | x | (x) |
| Diabetes | x | x | x | (x) |
| Obstructive sleep apnea | x | x | x | (x) |
| Chronic obstructive pulmonary disease | x | x | x | (x) |
| Smoking(yes/no/missing) | x | x | x | (x) |
| BMI (missing, spline with 4 knots) | x | x | x | (x) |
| **Other indications** |  |  |  |  |
| Pancreatitis | x | x | x | (x) |
| Use of thiazides | x | x | x | (x) |
| Use of beta-blockers | x | x | x | (x) |
| Use of oral estrogen replacement | x | x | x | (x) |
| Use of antipsychotics | x | x | x | (x) |
| **Contacts with health care in the prior year** |  |  |  |  |
| Hospitalized (yes/no) | x |  | x | (x) |
| Out-patient contacts (yes/no, number of contacts, modelled as spline with 4 knots) | x |  | x | (x) |
| Medication use (yes/no, number of different  ATCs-codes filled, modelled as a spline with 4 knots) | x |  | x | (x) |
| **Predicted pre-breast cancer LDL-cholesterol level** |  |  | x |  |
| **Predicted probability of pre-breast cancer LDL-cholesterol measurement** |  | weight | x | weight |
| **Predicted probability of post-breast cancer LDL-cholesterol measurement** |  |  |  | weight |

Abbreviations: AIT aromatase inhibitor treatment, ASCVD atherosclerotic cardiovascular disease, ATC Anatomical Therapeutic Chemical, BMI body mass index, LDL-cholesterol low density lipoprotein-cholesterol, HER2 human epidermal growth factor receptor 2; x variable included in the respective model, (x) variable included in a sensitivity analysis

# **Table S5. Distribution of additional covariates according to use of AIT^a^**

| **Characteristics** | **Use of AIT** | | **No use of AIT** | |
| --- | --- | --- | --- | --- |
|  | **n** | **%** | **n** | **%** |
| **Overall** | 7919 | 100.0% | 2542 | 100.0% |
| **Demographic characteristics at index date** |  |  |  |  |
| Region of residence |  |  |  |  |
| Capital Region | 2795 | 35.3% | 997 | 39.2% |
| Zealand | 1294 | 16.3% | 348 | 13.7% |
| Southern Denmark | 707 | 8.9% | 188 | 7.4% |
| Central Denmark | 2242 | 28.3% | 747 | 29.4% |
| Northern Denmark | 881 | 11.1% | 262 | 10.3% |
| **Indications for measurement of lipid profile (medical history)** |  |  |  |  |
| Lipid lowering drugs | 3044 | 38.4% | 986 | 38.8% |
| Low-intensity statin | 124 | 1.6% | 39 | 1.5% |
| Moderate-intensity statin | 2241 | 28.3% | 718 | 28.2% |
| High-intensity statin or  statin in combination | 625 | 7.9% | 209 | 8.2% |
| Other lipid-lowering  drugs | 54 | 0.7% | 20 | 0.8% |
| Any CVD | 4213 | 53.2% | 1345 | 52.9% |
| Ischemic heart disease | 907 | 11.5% | 323 | 12.7% |
| Stroke | 350 | 4.4% | 122 | 4.8% |
| Heart failure | 219 | 2.8% | 82 | 3.2% |
| Essential hypertension | 3833 | 48.4% | 1171 | 46.1% |
| Familial disposition to premature ASCVD | 655 | 8.3% | 207 | 8.1% |
| Impaired renal function | 720 | 9.1% | 227 | 8.9% |
| Diabetes | 1113 | 14.1% | 347 | 13.7% |
| Obstructive sleep apnea | 60 | 0.8% | 10 | 0.4% |
| Chronic obstructive pulmonary disease | 439 | 5.5% | 158 | 6.2% |
| Pancreatitis | 73 | 0.9% | 19 | 0.7% |
| Use of thiazides | 2375 | 30.0% | 712 | 28.0% |
| Use of beta-blockers | 1550 | 19.6% | 443 | 17.4% |
| Use of oral estrogen replacement | 1833 | 23.1% | 565 | 22.2% |
| Use of antipsychotics | 269 | 3.4% | 77 | 3.0% |
| BMI |  |  |  |  |
| ≤18.5 | 90 | 1.1% | 46 | 1.8% |
| >18.5-25 | 2391 | 30.2% | 910 | 35.8% |
| >25-30 | 2068 | 26.1% | 677 | 26.6% |
| >30-35 | 1043 | 13.2% | 318 | 12.5% |
| >35-40 | 389 | 4.9% | 109 | 4.3% |
| ≥40 | 150 | 1.9% | 53 | 2.1% |
| Missing | 1788 | 22.6% | 429 | 16.9% |
| Smoking |  |  |  |  |
| Yes | 917 | 11.6% | 299 | 11.8% |
| No | 3895 | 49.2% | 1250 | 49.2% |
| Missing | 3107 | 39.2% | 993 | 39.1% |
| **Contacts with health care within 1 year prior to index date^b^** |  |  |  |  |
| ≥1 overnight in-patient contact | 1522 | 19.2% | 552 | 21.7% |
| No. of out-patient contacts [median, (IQR)] | 5(4,8) |  | 5(4,8) |  |
| No. of different drugs used [median, (IQR)] | 6(4,9) |  | 6(4,10) |  |

Abbreviations: AIT aromatase inhibitor treatment, ASCVD atherosclerotic cardiovascular disease, BMI body mass index, CVD cardiovascular disease, HER2 human epidermal growth factor receptor 2, IQR interquartile range

^a ‘^Use of AIT’ is defined as allocated and dispensed treatment with AIT, and ‘No use of AIT’ is defined as no allocated and no dispensed treatment with AIT; both as recorded in the clinical database of the Danish Breast Cancer Group (see Table S3 for further definitions).

^b^ Index date defined as date of breast cancer diagnosis (defined as surgery date or biopsy date if neoadjuvant treatment) as recorded in the clinical database of the Danish Breast Cancer Group.

# **Figure S3. Distribution of the included pre-breast cancer and post-breast cancer LDL-cholesterol measurements in the two time windows in a nationwide cohort of postmenopausal women with early breast cancer with and without use of AIT, January 2009 to December 2020, Denmark**


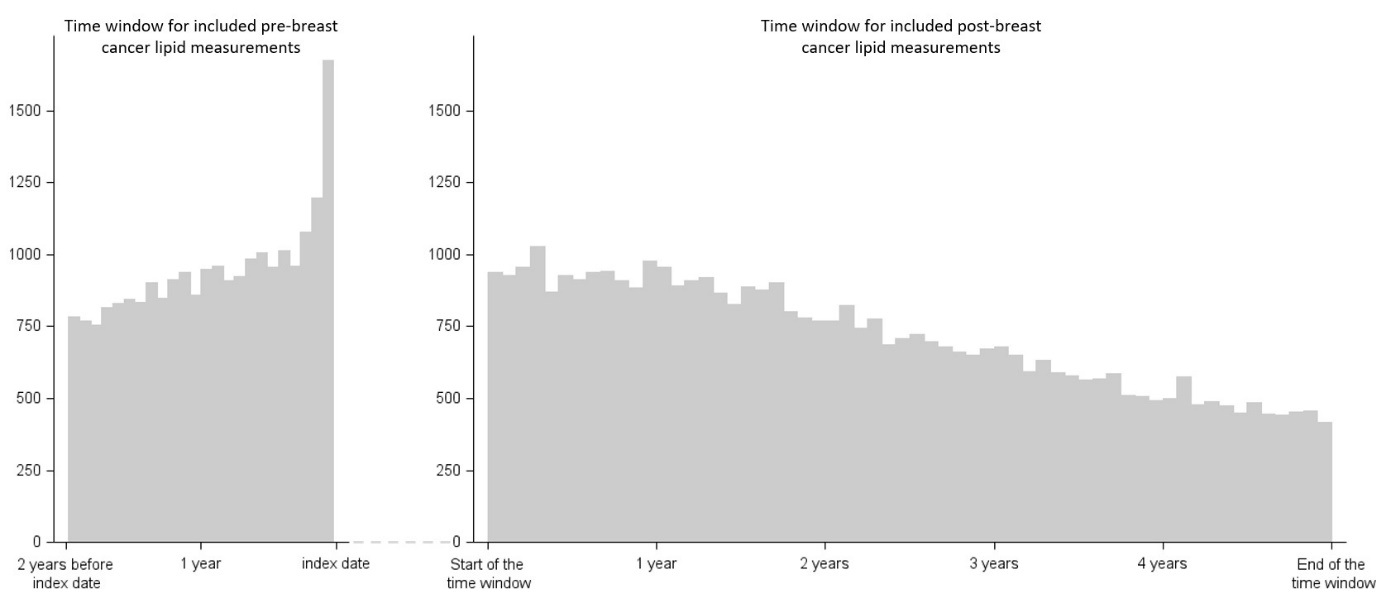


Abbreviations: AIT aromatase inhibitor treatment, LDL low-density lipoprotein

| **Table S6. Difference in LDL-cholesterol-change according to use of AIT^a^ in a nationwide cohort of postmenopausal women with early breast cancer, January 2009 to December 2020, Denmark, analyses with stratification according to subgroups^b^** | | | | | | | |
| --- | --- | --- | --- | --- | --- | --- | --- |
|  | **Women** | **Pre-breast cancer LDL-cholesterol^c^** | **Post-breast cancer LDL-cholesterol** | | **LDL-cholesterol-change** | **Difference in LDL-cholesterol-change** | |
|  | **n** | **mean**  **(mmol/L)** | **n** | **mean (mmol/L)** | **mean**  **(mmol/L)** | **crude**  **(mmol/L)** | **adjusted^d^**  **(mmol/L)** |
| **Timing of the post-breast cancer lipid-measurements by years since start of time window^e^** | | | | | | | |
| <1 year |  |  |  |  |  |  |  |
| No use of AIT | 1689 | 2.90 | 2651 | 2.81 | -0.09 | 0 ref. | 0 ref. |
| Use of AIT | 5468 | 2.89 | 8554 | 2.79 | -0.09 | -0.01(-0.05,0.03) | -0.02(-0.07,0.02) |
| 1-2 years |  |  |  |  |  |  |  |
| No use of AIT | 2014 | 2.95 | 4474 | 2.79 | -0.16 | 0 ref. | 0 ref. |
| Use of AIT | 6435 | 2.92 | 14487 | 2.75 | -0.17 | -0.03(-0.07,0.001) | -0.05(-0.09,-0.01) |
| 3-4 years |  |  |  |  |  |  |  |
| No use of AIT | 1393 | 3.00 | 3085 | 2.75 | -0.25 | 0 ref. | 0 ref. |
| Use of AIT | 4342 | 2.96 | 9499 | 2.69 | -0.28 | -0.01(-0.05,0.03) | -0.03(-0.07,0.02) |
| **History of ischemic CVD^b^** | | | | |  |  |  |
| *No history of IHD or IS* | |  |  |  |  |  |  |
| No use of AIT | 2123 | 3.04 | 8319 | 2.90 | -0.15 | 0 ref. | 0 ref. |
| Use of AIT | 6738 | 3.01 | 26887 | 2.84 | -0.17 | -0.03(-0.06,0.003) | -0.04(-0.08,-0.004) |
| *History of IHD or IS* | |  |  |  |  |  |  |
| No use of AIT | 419 | 2.58 | 1891 | 2.41 | -0.17 | 0 ref. | 0 ref. |
| Use of AIT | 1181 | 2.53 | 5653 | 2.40 | -0.13 | 0.04(-0.04,0.11) | 0.02(-0.05,0.10) |
| **Age^b^** | | | |  |  |  |  |
| *<70 years* | |  |  |  |  |  |  |
| No use of AIT | 1704 | 3.05 | 6978 | 2.90 | -0.15 | 0 ref. | 0 ref. |
| Use of AIT | 4496 | 3.04 | 18620 | 2.86 | -0.18 | -0.03(-0.07,0.005) | -0.05(-0.09,-0.01) |
| *≥70 years* |  |  |  |  |  |  |  |
| No use of AIT | 838 | 2.79 | 3232 | 2.65 | -0.14 | 0 ref. | 0 ref. |
| Use of AIT | 3423 | 2.81 | 13920 | 2.67 | -0.14 | -0.003(-0.05,0.05) | -0.003(-0.06,-0.05) |
| **Use of lipid lowering drugs^b^** | | |  |  |  |  |  |
| *Lipid lowering drugs* | |  |  |  |  |  |  |
| No use of AIT | 986 | 2.45 | 4595 | 2.32 | -0.13 | 0 ref. | 0 ref. |
| Use of AIT | 3044 | 2.41 | 14407 | 2.28 | -0.12 | -0.01(-0.05,0.04) | -0.02(-0.08,0.03) |
| *No lipid lowering drugs* | |  |  |  |  |  |  |
| No use of AIT | 1556 | 3.30 | 5615 | 3.13 | -0.16 | 0 ref. | 0 ref. |
| Use of AIT | 4875 | 3.27 | 18133 | 3.08 | -0.19 | -0.03(-0.06,0.01) | -0.04(-0.09,-0.0001) |
| **History of hypertension^b^** | |  |  |  |  |  |  |
| *No hypertension* |  |  |  |  |  |  |  |
| No use of AIT | 1371 | 3.13 | 4920 | 2.99 | -0.14 | 0 ref. | 0 ref. |
| Use of AIT | 4086 | 3.11 | 15142 | 2.94 | -0.17 | -0.03(-0.07,0.01) | -0.05(-0.09,-0.001) |
| *Hypertension* |  |  |  |  |  |  |  |
| No use of AIT | 1171 | 2.78 | 5290 | 2.61 | -0.16 | 0 ref. | 0 ref. |
| Use of AIT | 3833 | 2.76 | 17398 | 2.60 | -0.16 | -0.003(-0.05,0.04) | -0.02(-0.07,0.03) |
| **History of diabetes^b^** |  |  |  |  |  |  |  |
| *No diabetes* |  |  |  |  |  |  |  |
| No use of AIT | 2195 | 3.06 | 8262 | 2.92 | -0.14 | 0 ref. | 0 ref. |
| Use of AIT | 6806 | 3.04 | 26325 | 2.88 | -0.16 | -0.02(-0.05,0.01) | -0.03(-0.07,0.004) |
| *Diabetes* |  |  |  |  |  |  |  |
| No use of AIT | 347 | 2.35 | 1948 | 2.17 | -0.18 | 0 ref. | 0 ref. |
| Use of AIT | 1113 | 2.31 | 6215 | 2.13 | -0.18 | -0.02(-0.09,0.06) | -0.03(-0.11,0.05) |

Abbreviations: AIT Aromatase inhibitor treatment, BMI body mass index, CVD cardiovascular disease, IHD ischemic heart disease, IS ischemic stroke, LDL low-density lipoprotein.

^a ‘^Use of AIT’ is defined as allocated and dispensed treatment with AIT, and ‘No use of AIT’ is defined as no allocated and no dispensed treatment with AIT; both as recorded in the clinical database of the Danish Breast Cancer Group (see Table S3 for further definitions).

^b^ Subgroup categorizations are assessed at index date (date of breast cancer diagnosis defined as surgery date or biopsy date if neoadjuvant treatment). See Table S3 for further definitions.

^c^ The pre-breast cancer LDL-cholesterol level is predicted using a linear effects model for all women with at least one LDL-cholesterol-measurement prior to breast cancer diagnosis (see Supplementary Methods for further details); the difference between the mean of the observed LDL-cholesterol-levels prior to breast cancer diagnosis and the predicted one was <0.02mmol/L.

^d^ The p-values (testing for homogeneity) were as follows: time since start of the time window for included post-breast cancer lipid-measurements 0.31, history of ischemic CVD 0.10, age at baseline 0.17, lipid lowering drugs 0.50, hypertension 0.35, and diabetes 0.95.

^e^ Years since start of the time window for included post-breast cancer lipid-measurements reflects the timing of the post-breast cancer LDL-cholesterol measurement. The time window for included post-breast cancer lipid measurements started 3 months after surgery for breast cancer or end of chemotherapy whichever came last.

# **Table S7. Difference in LDL-cholesterol-change according to use of AIT^a^ in a nationwide cohort of postmenopausal women with early breast cancer, January 2009 to December 2020, Denmark, sensitivity analyses**

|  | **Women** | **Pre-breast cancer LDL-cholesterol^b^** | **Post-breast cancer LDL-cholesterol** | | **LDL-cholesterol-change** | **Difference in LDL-cholesterol-change** | |
| --- | --- | --- | --- | --- | --- | --- | --- |
| **Sensitivity analyses** | **n** | **mean**  **(mmol/L)** | **n** | **mean (mmol/L)** | **mean**  **(mmol/L)** | **crude**  **(mmol/L)** | **adjusted**  **(mmol/L)** |
| **Alternative definitions of exposure** | | | | | | | |
| *Exposure (use of AIT) updated during the time window for included post-breast cancer lipid measurements^c^* | | | | | | | |
| Never use of AIT | 2524 | 2.97 | 10073 | 2.82 | -0.15 | 0 ref. | 0 ref. |
| Current use of AIT | 7366 | 2.93 | 27001 | 2.78 | -0.15 | 0.001(-0.03,0.03) | -0.03(-0.07,0.005) |
| Past use of AIT | 2240 | 2.96 | 5539 | 2.74 | -0.22 | -0.13(-0.16,-0.09) | -0.04(-0.08,0.004) |
| *Time since AIT cessation* | | | | |  |  |  |
| Never use of AIT | 2524 | 2.97 | 10073 | 2.82 | -0.15 | 0 ref. | 0 ref. |
| Current use of AIT | 7366 | 2.93 | 27001 | 2.78 | -0.15 | 0.002(-0.03,0.03) | -0.03(-0.07,0.004) |
| Cessation of AIT^d^ |  |  |  |  |  |  |  |
| <1 year | 1769 | 2.93 | 2612 | 2.72 | -0.21 | -0.10(-0.14,-0.06) | -0.06(-0.11,-0.02) |
| 1-2 years | 1194 | 3.00 | 2417 | 2.78 | -0.23 | -0.14(-0.18,-0.09) | -0.01(-0.06,0.04) |
| 3+ years | 338 | 3.06 | 510 | 2.77 | -0.30 | -0.24(-0.31,-0.17) | -0.001(-0.07,0.07) |
| *Exposure defined as allocation to AIT* | | | |  |  |  |  |
| Allocation to AIT | 2622 | 2.96 | 10531 | 2.82 | -0.15 | 0 ref. | 0 ref. |
| No allocation to AIT | 9196 | 2.94 | 37131 | 2.78 | -0.16 | -0.01(-0.04,0.01) | -0.03(-0.06,0.01) |
| **Additional criteria for end of the time window for included post-breast cancer lipid measurements** | | | | | | | |
| *First use of tamoxifen* | | |  |  |  |  |  |
| No use of AIT | 2539 | 2.97 | 10185 | 2.82 | -0.15 | 0 ref. | 0 ref. |
| Use of AIT | 7690 | 2.93 | 30720 | 2.78 | -0.15 | -0.01(-0.04,0.02) | -0.02(-0.06,0.02) |
| *Diagnosis of contra-lateral, recurrent breast cancer, or other cancer* | | | | |  |  |  |
| No use of AIT | 2410 | 2.97 | 9174 | 2.82 | -0.15 | 0 ref. | 0 ref. |
| Use of AIT | 7580 | 2.94 | 29291 | 2.78 | -0.16 | -0.02(-0.05,0.01) | -0.03(-0.07,0.01) |
| **Additional exclusion criteria** | | | |  |  |  |  |
| *Exclusion of LDL-cholesterol measurements where the triglyceride-level was >4.5 mmol/L* | | | | | |  |  |
| No use of AIT | 2541 | 2.97 | 10192 | 2.82 | -0.15 | 0 ref. | 0 ref. |
| Use of AIT | 7914 | 2.94 | 32402 | 2.78 | -0.16 | -0.02(-0.05,0.01) | -0.03(-0.07,0.003) |
| **Analysis to evaluate selection bias** | | | | |  |  |  |
| *Including only one post-breast cancer LDL-cholesterol measurement (random)* | | | | |  |  |  |
| No use of AIT | 2542 | 2.97 | 2542 | 2.82 | -0.14 | 0 ref. | 0 ref. |
| Use of AIT | 7919 | 2.94 | 7919 | 2.77 | -0.17 | -0.02(-0.06,0.01) | -0.03(-0.08,0.01) |
| *Excluding patients who received neoadjuvant treatment* | | | | | | | |
| No use of AIT | 2354 | 2.96 | 9687 | 2.82 | -0.15 | 0 ref. | 0 ref. |
| Use of AIT | 7692 | 2.94 | 31737 | 2.78 | -0.16 | -0.02(-0.05,0.01) | -0.04(-0.08,-0.003) |
| **Further adjustment** | | |  |  |  |  |  |
| *Cardiovascular variables^e^* | |  |  |  |  |  |  |
| No use of AIT | 2542 | 2.97 | 10210 | 2.82 | -0.15 | 0 ref. | 0 ref. |
| Use of AIT | 7919 | 2.94 | 32540 | 2.78 | -0.16 | -0.02(-0.05,0.01) | -0.04(-0.07,0.001) |
| *Indication for LDL-cholesterol measurement^f^* | | |  |  |  |  |  |
| No use of AIT | 2542 | 2.97 | 10210 | 2.82 | -0.15 | 0 ref. | 0 ref. |
| Use of AIT | 7919 | 2.94 | 32540 | 2.78 | -0.16 | -0.02(-0.05,0.01) | -0.04(-0.07,0.001) |
| *Health care utilization^g^* | | |  |  |  |  |  |
| No use of AIT | 2542 | 2.97 | 10210 | 2.82 | -0.15 | 0 ref. | 0 ref. |
| Use of AIT | 7919 | 2.94 | 32540 | 2.78 | -0.16 | -0.02(-0.05,0.01) | -0.03(-0.07,0.01) |
| *BMI^h^* |  |  |  |  |  |  |  |
| No use of AIT | 2113 | 2.98 | 8567 | 2.83 | -0.15 | 0 ref. | 0 ref. |
| Use of AIT | 6131 | 2.95 | 25730 | 2.79 | -0.16 | -0.02(-0.05,0.01) | -0.03(-0.07,0.01) |
| *Smoking^h^* |  |  |  |  |  |  |  |
| No use of AIT | 1549 | 2.96 | 1549 | 2.82 | -0.15 | 0 ref. | 0 ref. |
| Use of AIT | 4812 | 2.95 | 4812 | 2.79 | -0.16 | -0.01(-0.05,0.02) | -0.03(-0.07,0.02) |
| **Further adjustment for time-dependent variables** | | | | |  |  |  |
| *Lipid lowering drugs* | |  |  |  |  |  |  |
| No use of AIT | 2542 | 2.97 | 10210 | 2.82 | -0.15 | 0 ref. | 0 ref. |
| Use of AIT | 7919 | 2.94 | 32540 | 2.78 | -0.16 | -0.02(-0.05,0.01) | -0.03(-0.06,0.01) |

| Abbreviations: AIT Aromatase inhibitor treatment, ATC anatomic therapeutic chemical classification, BMI body mass index, CVD cardiovascular disease, IHD ischemic heart disease, IS ischemic stroke, LDL low density lipoprotein.  ^a ‘^Use of AIT’ is defined as allocated and dispensed treatment with AIT, and ‘No use of AIT’ is defined as no allocated and no dispensed treatment with AIT; both as recorded in the clinical database of the Danish Breast Cancer Group (see Table S3 for further definitions).  ^b^ The pre-breast cancer LDL-cholesterol level is predicted using a linear effects model for all women with at least one LDL-cholesterol-measurement prior to breast cancer diagnosis (see Supplementary Methods for further details); the difference between the mean of the observed LDL-cholesterol-levels prior to breast cancer diagnosis and the predicted one was <0.02mmol/L.  ^c^ Among women not allocated to AIT to whom AIT is first dispensed during the lipid observational window, the LDL-cholesterol measurements following the date of first dispensed AIT are excluded.  ^d^ The p-value for the test of a difference according to time since AIT cessation was 0.02.  ^e^ Besides the variables included in the main model, we adjusted for pre-breast cancer lipid lowering treatment (no, low statin, moderate statin, high statin, statin in combination, other lipid lowering drugs), any CVD, ischemic heart disease, stroke, heart failure, essential hypertension, familial disposition to premature atherosclerotic cardiovascular disease, impaired renal function, diabetes, obstructive sleep apnea, chronic obstructive pulmonary disease.  ^f^ Besides the variables included in the main model, we adjusted for pancreatitis, use of thiazides, use of beta-blockers, use of oral estrogen replacement, and use of antipsychotics.  ^g^ Besides the variables included in the main model, we adjusted for hospitalization (yes/no), out-patient contacts (yes/no, number of contacts modeled as spline with 4 knots), medication use (yes/no, number of different ATC-codes filled, modeled as spline with 4 knots).  ^h^ This analysis is a complete-case analysis, i.e. only women with information about BMI, or smoking respectively, are included. The estimate, besides the covariates already included in the main model, is also adjusted for BMI or smoking. |
| --- |

# **Potential bias in relation to use of the register-based lipid-measurements**

In this study, we used laboratory values from a nationwide database which holds information about lipid levels collected as part of routine clinical care. Advantages to this include nationwide coverage (minimizes selection bias and maximizes power); however, presence of a lipid measurement may *per se* indicate a higher likelihood of an abnormal lipid-level. This can affect the chance of being included into the study, and it can affect the number of measurements among those included.

To be included in the study, women were required to be diagnosed with early breast cancer, be post-menopausal, and have at least one pre-breast cancer LDL-cholesterol measurement and one post-breast cancer LDL-cholesterol measurement. The measurement-requirements may limit the ability to make inferences to women with breast cancer who have not had LDL-cholesterol measured before and after breast cancer diagnosis. We addressed this in the final statistical model (model 4 described in further detail in Supplementary Methods) by including weights for the predicted probability of having an LDL-cholesterol measurement before and after breast cancer diagnosis, respectively, i.e. the women included in the final cohort (in Figure 1: n=10,461) are weighted to resemble the entire study population (in Figure 1: n=28,573 i.e. postmenopausal women diagnosed with breast cancer, Denmark, January 2009 to December 2020, eligible for inclusion in the study).

Besides the bias described above, residual bias may be related to the probability of having lipid-measurements for those women who fulfill the inclusion criteria for the study. If both AIT and the true LDL-cholesterol level affect the probability of having an LDL-cholesterol-measurement, then the association between AIT and LDL-cholesterol can be biased. The true LDL-cholesterol level is plausibly associated with the probability of having it measured. However, AIT is most likely not in our study. AIT assignment is based on known guideline factors that are included in the measurement probability weights in the statistical model, and AIT is not mentioned as an indication for monitoring lipid-levels in current Danish breast cancer guidelines ^2^. Moreover, results were similar when including only one (random) post-breast cancer LDL-cholesterol measurement (Table S7). Taken together, this makes it unlikely that differential probability of having lipid-measurements affects our conclusion.

# **Reference list**

1. Danish Breast Cancer Group. DBCGs protokoller, samlet oversigt. Accessed June 8, 2023. https://dbcg.dk/PDF/DBCG_2014_protokoller_oversigt_01.08.2014.pdf

2. Systemisk behandling af brystkræft - I – hvem skal anbefales adjuverende systemisk behandling? Version 1.3. Accessed October 8, 2023. https://www.dmcg.dk/Kliniske-retningslinjer/kliniske-retningslinjer-opdelt-paa-dmcg/brystcancer/systemisk-behandling-af-brystkraft---i--hvem-skal-anbefales-adjuverende-systemisk-behandling/

3. Pedersen CB. The Danish Civil Registration System. *Scandinavian Journal of Public Health*. 2011;39(7_suppl):22-25. doi:10.1177/1403494810387965

4. Jensen MB, Laenkholm AV, Offersen BV, et al. The clinical database and implementation of treatment guidelines by the Danish Breast Cancer Cooperative Group in 2007–2016. *Acta Oncologica*. 2018;57(1):13-18. doi:10.1080/0284186X.2017.1404638

5. Schmidt M, Schmidt SAJ, Sandegaard JL, Ehrenstein V, Pedersen L, Sørensen HT. The Danish National Patient Registry: a review of content, data quality, and research potential. *Clin Epidemiol*. 2015;7:449-490. doi:10.2147/CLEP.S91125

6. Pottegård A, Schmidt SAJ, Wallach-Kildemoes H, Sørensen HT, Hallas J, Schmidt M. Data Resource Profile: The Danish National Prescription Registry. *Int J Epidemiol*. 2017;46(3):798-798f. doi:10.1093/ije/dyw213

7. Laboratoriedatabasen - Sundhedsdatastyrelsen. Accessed November 9, 2023. https://sundhedsdatastyrelsen.dk/da/registre-og-services/om-de-nationale-sundhedsregistre/doedsaarsager-og-biologisk-materiale/laboratoriedatabasen

8. Hamilton S. Dokumentation af Laboratoriedatabasens Forskertabel Version 4. Accessed December 21, 2023. https://sundhedsdatastyrelsen.dk/-/media/sds/filer/registre-og-services/nationale-sundhedsregistre/doedsaarsager-og-biologisk-materiale/laboratoriedatabasen/dokumentation-af-labdatabasens-forskertabel.pdf?la=da

9. Jensen VM, Rasmussen AW. Danish education registers. *Scand J Public Health*. 2011;39(7_suppl):91-94. doi:10.1177/1403494810394715

10. Baadsgaard M, Quitzau J. Danish registers on personal income and transfer payments. *Scand J Public Health*. 2011;39(7_suppl):103-105. doi:10.1177/1403494811405098

11. Danmarks Statistik. Registre i Forskningsservices grunddatabank. Accessed March 11, 2024. https://www.dst.dk/extranet/forskningvariabellister/Oversigt%20over%20registre.html

12. Antonsen K, Rosenstock CV, Lundstrøm LH. The Danish Anaesthesia Database. *CLEP*. 2016;8:435-438. doi:10.2147/CLEP.S99517

13. Bjerregaard B, Larsen OB. The Danish Pathology Register. *Scand J Public Health*. 2011;39(7 Suppl):72-74. doi:10.1177/1403494810393563

14. Gjerstorff ML. The Danish Cancer Registry. *Scand J Public Health*. 2011;39(7 Suppl):42-45. doi:10.1177/1403494810393562

15. Danish Clinical Quality Program – National Clinical Registries (RKKP). Dokumentation af DBCG Kvalitetsdatabase for Brystkræft. Accessed September 28, 2024. https://www.rkkp-dokumentation.dk/Public/Databases.aspx?db=31&db2=1000000834

16. Engholm G, Ferlay J, Christensen N, et al. NORDCAN--a Nordic tool for cancer information, planning, quality control and research. *Acta Oncol*. 2010;49(5):725-736. doi:10.3109/02841861003782017

17. DCS arbejdsgruppe vedrørende præventiv kardiologi. Forebyggelse af hjertesygdom (opdateret 12. maj 2023)). Accessed September 1, 2023. https://nbv.cardio.dk/forebyggelse#342-primr-og-sekundr-forebyggelse

18. Hvidberg MF, Johnsen SP, Glümer C, Petersen KD, Olesen AV, Ehlers L. Catalog of 199 register-based definitions of chronic conditions. *Scand J Public Health*. 2016;44(5):462-479. doi:10.1177/1403494816641553

19. Grundy SM, Stone NJ, Bailey AL, et al. 2018 AHA/ACC/AACVPR/AAPA/ABC/ACPM/ADA/AGS/APhA/ASPC/NLA/PCNA Guideline on the Management of Blood Cholesterol: A Report of the American College of Cardiology/American Heart Association Task Force on Clinical Practice Guidelines. *Circulation*. 2019;139(25):e1082-e1143. doi:10.1161/CIR.0000000000000625

20. Pedersen RN, Öztürk B, Mellemkjær L, et al. Validation of an Algorithm to Ascertain Late Breast Cancer Recurrence Using Danish Medical Registries. *Clin Epidemiol*. 2020;12:1083-1093. doi:10.2147/CLEP.S269962
